# Single-cell transcriptomics reveals shared immunosuppressive landscapes of mouse and human neuroblastoma

**DOI:** 10.1136/jitc-2022-004807

**Published:** 2022-08-05

**Authors:** Ana Costa, Cécile Thirant, Amira Kramdi, Cécile Pierre-Eugène, Caroline Louis-Brennetot, Orphée Blanchard, Didier Surdez, Nadege Gruel, Eve Lapouble, Gaëlle Pierron, Deborah Sitbon, Hervé Brisse, Arnaud Gauthier, Paul Fréneaux, Mylène Bohec, Virginie Raynal, Sylvain Baulande, Renaud Leclere, Gabriel Champenois, Andre Nicolas, Didier Meseure, Angela Bellini, Aurelien Marabelle, Birgit Geoerger, Fatima Mechta-Grigoriou, Gudrun Schleiermacher, Laurie Menger, Olivier Delattre, Isabelle Janoueix-Lerosey

**Affiliations:** 1 Inserm U830, Equipe Labellisée LNCC, Diversity and Plasticity of Childhood Tumors Lab, PSL Research University, Institut Curie Research Centre, Paris, France; 2 SIREDO: Care, Innovation and Research for Children, Adolescents and Young Adults with Cancer, Institut Curie, Paris, France; 3 Department of Translational Research, Institut Curie, Paris, France; 4 Unité de Génétique Somatique, Institut Curie, Paris, France; 5 Department of Imaging, PSL Research University, Institut Curie, Paris, France; 6 Department of Biopathology, Institut Curie, Paris, France; 7 Genomics of Excellence (ICGex) Platform, Institut Curie, Paris, France; 8 Laboratory Recherche Translationnelle en Oncologie Pédiatrique (RTOP), Laboratoire “Gilles Thomas”, Institut Curie, Paris, France; 9 Inserm U1015 & CIC1428, Université Paris Saclay, Gustave Roussy, Villejuif, France; 10 Inserm U1015, Department of Pediatric and Adolescent Oncology, Université Paris-Saclay, Gustave Roussy, Villejuif, France; 11 Inserm U830, Equipe labelisée LNCC, Stress and Cancer Laboratory, PSL Research University, Institut Curie Research Centre, Paris, France; 12 Inserm U932, PSL Research University, Institut Curie, Paris, France

**Keywords:** Neuroblastoma, Tumor Microenvironment, Myeloid-Derived Suppressor Cells, Gene Expression Profiling, Macrophages

## Abstract

**Background:**

High-risk neuroblastoma is a pediatric cancer with still a dismal prognosis, despite multimodal and intensive therapies. Tumor microenvironment represents a key component of the tumor ecosystem the complexity of which has to be accurately understood to define selective targeting opportunities, including immune-based therapies.

**Methods:**

We combined various approaches including single-cell transcriptomics to dissect the tumor microenvironment of both a transgenic mouse neuroblastoma model and a cohort of 10 biopsies from neuroblastoma patients, either at diagnosis or at relapse. Features of related cells were validated by multicolor flow cytometry and functional assays.

**Results:**

We show that the immune microenvironment of MYCN-driven mouse neuroblastoma is characterized by a low content of T cells, several phenotypes of macrophages and a population of cells expressing signatures of myeloid-derived suppressor cells (MDSCs) that are molecularly distinct from the various macrophage subsets. We document two cancer-associated fibroblasts (CAFs) subsets, one of which corresponding to CAF-S1, known to have immunosuppressive functions. Our data unravel a complex content in myeloid cells in patient tumors and further document a striking correspondence of the microenvironment populations between both mouse and human tumors. We show that mouse intratumor T cells exhibit increased expression of inhibitory receptors at the protein level. Consistently, T cells from patients are characterized by features of exhaustion, expressing inhibitory receptors and showing low expression of effector cytokines. We further functionally demonstrate that MDSCs isolated from mouse neuroblastoma have immunosuppressive properties, impairing the proliferation of T lymphocytes.

**Conclusions:**

Our study demonstrates that neuroblastoma tumors have an immunocompromised microenvironment characterized by dysfunctional T cells and accumulation of immunosuppressive cells. Our work provides a new and precious data resource to better understand the neuroblastoma ecosystem and suggest novel therapeutic strategies, targeting both tumor cells and components of the microenvironment.

**What is already known on this topic**

- High-risk neuroblastoma is associated with a poor outcome despite multimodal therapies targeting tumor cells. No complete pictures of the immune landscape have been obtained so far in this pediatric cancer.

**What this study adds**

- This study characterizes for the first time the whole cellular composition of the neuroblastoma microenvironment without any prior assumptions on surface markers, both in a relevant and immunocompetent mouse neuroblastoma model and in a cohort of patients, and documents multiple features of immunosuppression.

**How this study might affect research, practice or policy**

- The new data resource, generated in this study through single-cell transcriptomics, brings additional knowledge to suggest novel therapies reversing the immunosuppressive tumor microenvironment in neuroblastoma patients.

## Introduction

Neuroblastoma is an embryonal cancer of the sympathetic nervous system observed in early childhood that accounts for 8%–10% of pediatric cancers.^1^ Even when metastatic, this cancer is characterized by diverse clinical behaviors, ranging from spontaneous regression to fatal outcome. Notably, one out of two children diagnosed with neuroblastoma presents with high-risk clinicobiological features and a 5-year overall survival rate below 40%. Multimodal therapies combine surgery, myeloablative chemotherapy, radiation therapy and immunotherapy with monoclonal antibodies targeting the disialoganglioside GD2, highly expressed at the cell surface of tumor cells.^2–5^

Tumor microenvironment (TME) has now been recognized as a key component of the tumor ecosystem and diverse cell types of the TME might promote or limit tumor outgrowth.^6^ Several approaches to boost autologous T cell activity against tumor cells or using engineered T cell immunotherapy have shown dramatic clinical responses.^7^ Yet, only a subset of patients with cancer durably experienced responses to current immunotherapies targeting T cell inhibitory checkpoint signaling pathways^8^ or using chimeric-antigen receptor T cells.^9 10^ The development of other strategies relying on immunotherapy targets beyond T cells requires a complete understanding of the TME complexity^11^ and, in particular, that of the tumor-infiltrating myeloid cell compartment.^12^

Some data suggest that the immune microenvironment of pediatric cancers might be different from adult cancers with less infiltration by T cells and dendritic cells, but with more NK cells, macrophages and other myeloid cells.^13^ Moreover, pediatric cancers in general and neuroblastoma in particular have a low rate of somatic mutations in their DNA as compared with adult cancers, except for rare outliers.^14^ This could lead to a lower rate of tumor-associated neoantigens and a lower immunogenicity of pediatric tumors compared with highly mutated adult ones. In neuroblastoma, some studies have started to explore the TME. Several insights have been obtained with the description of a higher infiltration of tumor-associated macrophages in non-*MYCN* amplified metastatic tumors as compared with loco-regional cases,^15^ the report of cancer-associated fibroblasts (CAFs),^16^ endothelial cells^17^ and inflammatory immune cells^18^ in the neuroblastoma TME. Defects in antigen-presenting machinery and low expression levels of MHC class I molecules by neuroblastoma cells have also been reported.^19^ Very recently, it has been shown that peptide-centric CARs can recognize unmutated tumor peptides across multiple HLA genotypes and that targeting the PHOX2B transcription factor with such CARs induces an upregulation of MHC and killing of neuroblastoma cells in vitro and in animal models.^20^

Until now, the characterization of the different populations of the neuroblastoma TME relied on limited numbers of markers. In this work, we combined single-cell transcriptomics with multicolor flow cytometry (FACS) and immunohistochemistry (IHC) approaches to provide a complete characterization of the TME without any prior assumptions on surface markers, first in the transgenic TH-MYCN neuroblastoma mouse model. This model has been widely used both for fundamental research and therapeutics studies as the developing tumors reproduce many features of human neuroblastoma in an immunocompetent background.^21 22^ TH-MYCN tumors arise spontaneously from sympathetic ganglia, they share most of the genetic abnormalities observed in *MYCN*-amplified tumors, and show histology and imaging characteristics highly similar to the human disease. Next, in a second step, single-cell transcriptomic data were generated for a cohort of 10 neuroblastoma patients studied at diagnosis or at relapse.

## Methods

TH*-*MYCN mice used in this study have been previously described.^21^ Neuroblastoma samples for single-cell analyses were obtained from patients treated at Institut Curie. Surplus tissues obtained at diagnosis or relapse were processed immediately after receipt at the laboratory for molecular diagnosis (Unité de Génétique Somatique). Written informed consents for this study, including the analysis of surplus tumor tissue were obtained for all patients from parents or guardians. Out of the 10 studied patients (online supplemental table 1), 4 patients were enrolled in the MICCHADO study (ClinicalTrials.gov identifier NCT03496402) and 4 patients in the MAPPYACTS trial (ClinicalTrials.gov identifier NCT02613962), with 2 patients enrolled in both programs. The technical details of single-cell dissociation, scRNA-seq analysis, immunohistochemistry, FACS analysis, myeloid-derived suppressor cells (MDSCs) isolation and T cell suppression assays are provided in online supplemental methods.

10.1136/jitc-2022-004807.supp3 Supplementary data

10.1136/jitc-2022-004807.supp2 Supplementary data

## Results

### Composition of TH-MYCN mouse tumors at the single-cell resolution

**Figure 1:**
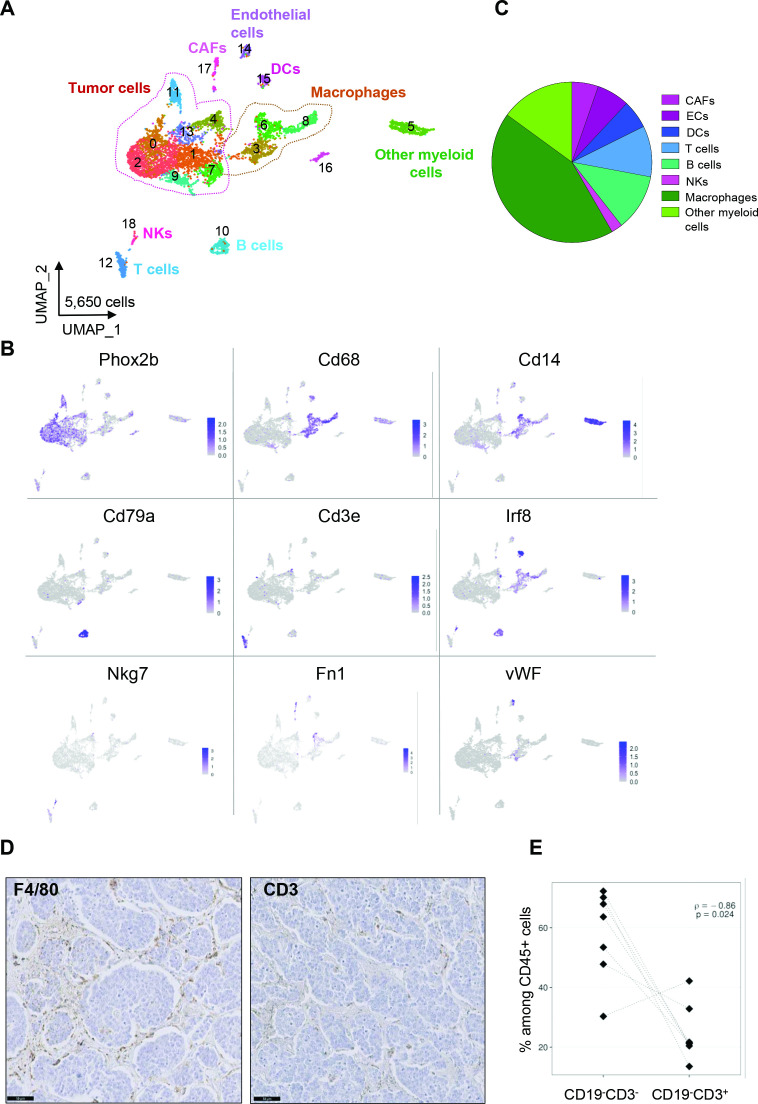
Deciphering the cellular ecosystem of the TH-MYCN mouse neuroblastoma model. (A) Uniform manifold approximation and projection (UMAP) of the 5650 cells obtained after the integration by Seurat of the three tumors. (B) Expression of markers in the UMAP analysis shown in (A) identifies tumor cells and distinct populations defining the TME. (C) Macrophages and other myeloid cells are a major component of the TME as shown by the repartition of the eight TME subpopulations identified by scRNA-seq. (D) Representative images of the staining of macrophages (F4/80) and T lymphocytes (CD3) by IHC on the same tumor. Scale bar: 50 µm. (E) Inverse correlation between the number of CD19^-^CD3^-^ cells corresponding to myeloid and NK cells and CD3^+^CD19^-^ cells being T cells obtained by FACS analysis on seven tumors and normalized to CD45^+^ cells. CAFs, cancer-associated fibroblasts; IHC, immunohistochemistry; TME, tumor microenvironment.

To obtain a complete overview of the cell populations composing MYCN-driven mouse neuroblastoma, three independent whole fresh tumors were analyzed by single-cell RNA-seq. Tumors were dissociated into single-cell suspensions, then cells were captured on the Chromium device (10X Genomics) and sequenced on the Novaseq (Illumina) sequencer (online supplemental figure 1A). After integration with Seurat, we obtained 5650 cells, with an average of 1883 cells per tumor (online supplemental figure 1B). Cluster analysis was performed using Seurat and cell populations were first annotated through the expression of canonical cell type gene markers. In total, eight cell types could be defined in addition to tumor cells represented by several clusters and defined by the expression of the Phox2b transcription factor (figure 1A, B). Macrophages (Cd68^+^), other myeloid cells (Cd14^+^), B cells (Cd79a^+^), T cells (Cd3e^+^), dendritic cells (Irf8^+^) and NK cells (Nkg7^+^) composed the immune microenvironment of these tumors. CAFs (Fn1^+^) and endothelial cells (vWF^+^) were also detected in these samples. Macrophages and Cd14^+^ myeloid cells were the most abundant TME populations (figure 1C). All annotated cell types were detected in each tumor individually at different proportions (online supplemental figure 1C).

10.1136/jitc-2022-004807.supp1 Supplementary data

In parallel, we performed IHC and FACS analyses on distinct tumors of the same model. First, IHC using F4/80 or CD3 antibodies confirmed that macrophages were more abundant in this model as compared with T lymphocytes (figure 1D). Interestingly, by FACS, we observed a strong anti-correlation between the population of CD3^-^CD19^-^ cells, corresponding to myeloid and NK cells and T lymphocytes (CD3^+^ CD19^-^) among CD45^+^ cells (figure 1E). These observations therefore suggested an immunosuppressive myeloid TME in TH-MYCN mouse tumors.

### Heterogeneity of macrophages in mouse neuroblastoma

**Figure 2:**
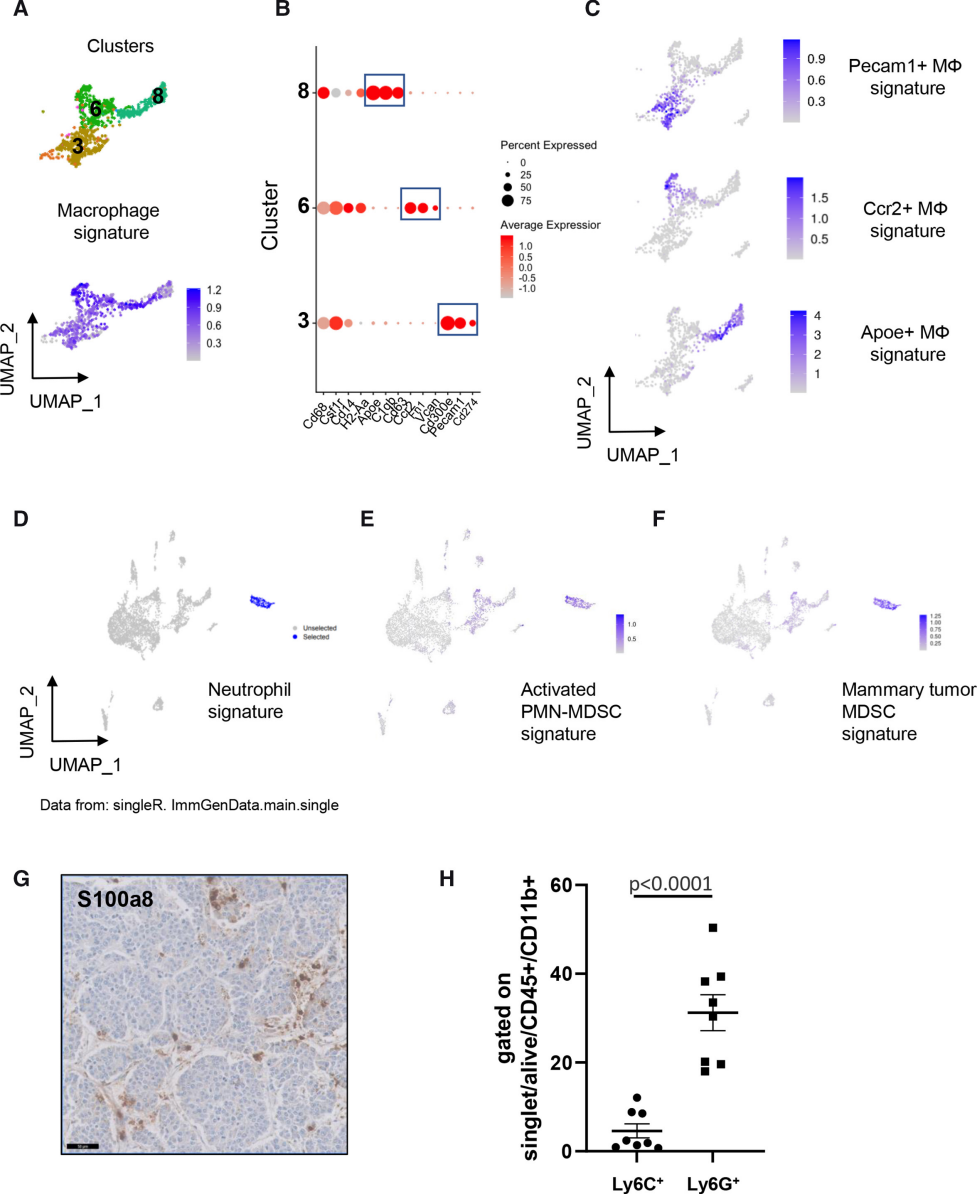
Macrophage heterogeneity and myeloid-derived suppressor cells (MDSCs) in TH-MYCN mouse tumors. (A) Uniform manifold approximation and projection (UMAP) analysis uncovers three different clusters of macrophages (top) expressing a common signature (bottom). (B) Dotplot showing the expression of marker genes highlighting the differences between three macrophage subsets. (C) Expression of the signatures of the three macrophage subsets. (D) SingleR identifies cluster 5 cells as neutrophils. (E) and (F) cluster 5 cells are highlighted by a signature of activated PMN-MDSCs^29^ and a signature of mammary tumor MDSCs.^31^ (G) Representative image of the staining of S100A8 by IHC obtained on the same tumor as the one showed in figure 1D. Scale bar: 50 µm. (H) Percentage of cells expressing Ly6C and Ly6G by FACS among CD45^+^CD11b^+^ cells in 8 TH-MYCN tumors. FACS, multicolor flow cytometry; IHC, immunohistochemistry; PMN, polymorphonuclear neutrophils.

As shown in figure 1A, three clusters of macrophages (clusters 3, 6 and 8) which all expressed a common signature including Cd68, Csf1r, Ccr2, Cd86, Adgre1 (encoding F4/80) and Lgals3 were detected in the TME of MYCN-driven tumors (figure 2A). These three macrophage clusters could be further defined by specific markers as follows: cluster 3 was characterized by high expression of Pecam1 and Cd300e; cluster 6 exhibited a high expression level of Ccr2 and Fn1 whereas cluster 8 strongly expressed Apoe, C1qb and Cd63 (figure 2B, online supplemental figure 2A, online supplemental table 2). These clusters were respectively named Pecam1^+^, Ccr2^+^and Apoe^+^macrophages. A classification of macrophages has been previously used to distinguish ‘classically activated’ M1 and ‘alternatively activated’ M2 macrophages in response to defined stimuli in vitro and respectively associated with antitumor and protumor activity.^23^ Signatures have also been proposed to identify angiogenesis and phagocytosis associated phenotypes in macrophages^24^ (online supplemental table 3). We, therefore, evaluated these distinct signatures defined in human, on our murine data. This analysis revealed that Apoe^+^ macrophages expressed signatures associated with M2 and phagocytosis phenotypes (online supplemental figure 2B). The Ccr2^+^ and Pecam1^+^ clusters were not highlighted by any of these signatures. These observations are in line with recently published data demonstrating that macrophages exhibit high heterogeneity in different tumor types.^24^ We next generated short signatures including a reduced number of genes to detect these three macrophage subsets. A signature including *Cd300e*, *Cd82*, *Pecam1*, *Il10*, *Cd274*, *Pglyrp1* and *Pag1* characterized the Pecam1^+^ macrophages, whereas a signature including *Ccr2*, *Sell*, *Vcan*, *Ly6c2*, *Fn1* and *F13a1* identified Ccr2^+^ macrophages; a three-gene signature (*Apoe*, *C1qb* and *Cd63*) defined Apoe^+^ macrophages as shown in the uniform manifold approximation and projection visualization (figure 2C). Interestingly, *Tgfb1,* known to have a central role in the TME immunosuppression^25^ was highly expressed in macrophages, regardless of the subset (online supplemental figure 2C).

### The TH-MYCN mouse TME contains a population of MDSCs

We next focused on cluster 5 highly expressing Cd14 (figure 1A, B). When applying SingleR using annotations for mouse cell populations,^26^ cluster 5 was strongly labeled with a neutrophil signature (figure 2D). Cells from this cluster exhibited high levels of *S100a8*, *S100a9* and *Mmp9*, but showed an absent or low expression of *Cd68* and *H2-Aa*, that are typical markers of macrophages (online supplemental figure 3A). S100a8 (Calgranulin-A) and S100a9 (Calgranulin-B) are calcium-binding and zinc-binding proteins known to form a stable heterodimer called calprotectin that have prominent role in the regulation of inflammatory processes and immune response. Together with Mmp9, S100a8 and S100a9 are known to be highly expressed by neutrophils. These observations suggested that cells from cluster 5 may correspond to tumor-associated neutrophils which have recently emerged as an important component of the TME.^27 28^

Strikingly, cluster 5 cells also expressed a gene signature defining activated PolyMorphoNuclear neutrophils-MDSCs (PMN-MDSCs) (figure 2E), described in mouse tumors, exhibiting a high expression of S100a8/a9 and characterized by a potent immune suppressive activity.^29^ A low expression of a signature of PMC-MDSCs was observed only in a minor fraction of cluster 5 (online supplemental figure 3B). MDSCs constitute a heterogeneous population of myeloid cells that are pathologically activated and have immunosuppressive properties; they are now recognized as major regulators of immune responses in cancer.^30^ MDSCs include two major subsets based on their phenotypic features: PMN-MDSCs (also called granulocytic (G)-MDSCs) and monocytic (M)-MDSCs.^30^ PMNs have been implicated in antitumor activity and expression signatures have been recently defined that distinguish activated PMN-MDSCs from PMN-MDSCs.^29^ Interestingly, cluster 5 cells were also characterized by strong expression of a signature common to both PMN-MDSCs and M-MDSCs, recently identified in the mouse MMTV-PyMT mammary tumor model^31^ (figure 2F). The concomitant expression of signatures of both neutrophils and PMN-MDSCs by cluster 5 fits with the recent idea that PMN-MDSCs correspond to a neutrophil population with immunosuppressive properties^27 28^ and that PMN-MDSCs and activated PMN-MDSCs represent two populations of neutrophils in tumor-bearing mice.^29^ Of interest, cluster 5 cells strongly expressed *Il1b* and *Arg2* (online supplemental figure 3A) that are known to be involved in immunosuppression.^32 33^ A subset of these cells also expressed Csf-1 (colony-stimulating factor-1), which may promote macrophage accumulation within tumors and regulate macrophage survival, proliferation and differentiation.^34^

IHC experiments on sections of MYCN-driven mouse tumors confirmed the infiltration of MDSCs highly expressing S100a8 in the tumors (figure 2G). Next, tumors were analyzed by FACS using cell-surface markers defining PMN-MDSCs and M-MDSCs in mice, with PMN-MDSCs characterized by a CD11b^+^Ly6G^+^Ly6C^low^ phenotype and M-MDSCs being CD11b^+^Ly6G^-^Ly6C^high^ cells^35^(online supplemental figure 4). As shown in figure 2H, both populations were detected in a series of 8 independent tumors and PMN-MDSCs were more abundant compared with M-MDSC cells. Altogether, these data indicate that cluster 5 cells of TH-MYCN tumors represent a population of immunosuppressive myeloid cells compatible with activated PMN-MDSCs and representing a particular subset of neutrophils.

### Two subsets of CAFs are defined in mouse neuroblastoma

In many adult cancers, CAFs have been shown to constitute an abundant component of the TME.^36^ In neuroblastoma, CAFs remain poorly characterized. Our single-cell analysis of murine tumors revealed a cluster of CAFs (cluster 17) characterized by *Fn1* expression (figure 1A, B) that could further be divided into two subclusters when increasing the resolution in the clustering analysis (online supplemental figure 5A). Using signatures of CAFs defined in human breast and ovarian cancers, one subcluster showed a high expression of a CAF-S1 signature, whereas the other one had a strong signal for a CAF-S4 signature (online supplemental figure 5B).^37^ CAF-S1 and CAF-S4 have been shown to have immunosuppressive function and pro-metastatic function, respectively.^36 37^ The CAF-S1 subcluster also mildly expressed a iCAF (inflammatory CAFs) signature^38^ whereas the CAF-S4 subcluster was also highlighted by signatures of stromal cells called perivascular-like (PVL) cells, being either differentiated-PVLs (dPVL*—Tagln*, *Cd9*, *Mylk* and *Cnn1*) and immature PVLs (imPVL*—Cd36*, *Notch3*, *Rgs5*, *Rhob* and *Itga1*) (online supplemental figure 5B).^39^ A differential analysis between the CAF-S1 and CAF-S4 clusters identified upregulated genes in each subset (online supplemental figure 5C and 5D). The two CAF populations were then validated by FACS (online supplemental figure 5C). Interestingly, our data revealed that several chemokines including Ccl2, Cxcl1 and Cxcl12 that have been shown to contribute to immunosuppressive TME are highly expressed by CAF-S1 cells (online supplemental figure 5F).

### Single-cell transcriptomics reveals a variety of myeloid cells in the TME of human neuroblastoma

Next, we performed single-cell transcriptomic analysis on 10 biopsies of human neuroblastoma obtained at diagnosis or at relapse (online supplemental table 1). The integration of the 10 biopsies (n=20,296 cells) highlighted tumor cells (*PHOX2B*^+^, *GATA3*^+^) as well as several populations of the TME (online supplemental figure 6A and 6B). Analysis using the InferCNV tool confirmed that tumor cells exhibited the emblematic genetic alterations of neuroblastoma such as 17q gain (online supplemental figure 6C). In contrast, no such alteration could be detected in the various TME clusters.

**Figure 3:**
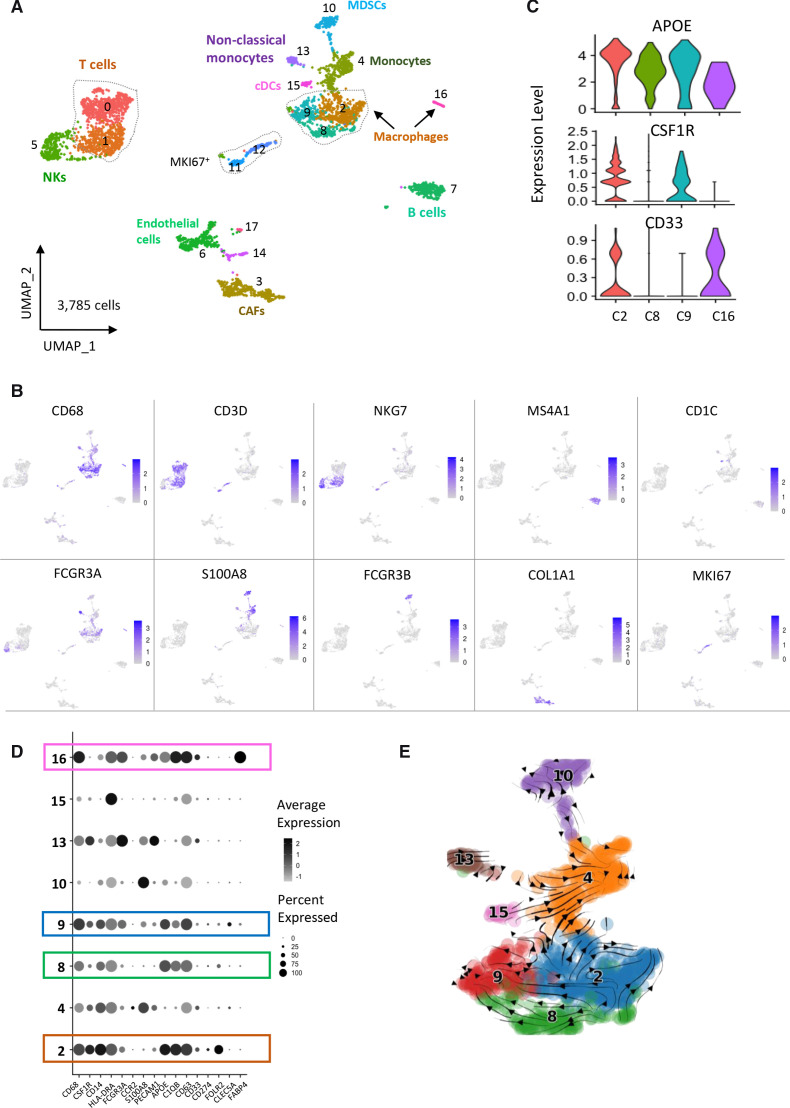
Characterization of the TME in a cohort of 10 neuroblastoma biopsies by single-cell transcriptomic analysis. (A) UMAP of 3785 cells obtained after the integration of the 10 biopsies and clustering of non-tumor cells only. Tumor cells were defined by the expression of *PHOX2B* and presence of genomic alterations inferred from scRNA-seq data. (B) Cell types were determined by canonical marker genes. (C) Violin plots showing the expression of *APOE*, *CSF1R* and *CD33* that defines three different macrophages subsets. (D) Dotplot showing the expression of genes defining the different myeloid cell populations. (E) scVelo analysis indicating that macrophages from clusters 8 and 9 likely derive from macrophages of cluster 2. CAFs, cancer-associated fibroblasts; TME, tumor microenvironment; UMAP, uniform manifold approximation and projection.

To better discriminate the various TME populations, we then used Harmony to integrate the 10 biopsies after excluding tumor cells (figure 3A, online supplemental figure 6D). Expression of canonical cell type gene markers combined with SingleR analysis for human cells allowed to identify the following populations of immune cells: macrophages (*CD68^+^*, clusters 2, 8, 9 and 16), T cells (*CD3D^+^,* clusters 0 and 1), NK cells (*NKG7^+^*, cluster 5), B cells (*MS4A1^+^*encoding CD20, cluster 7), conventional dendritic cells (*CD1C*^+^, cluster 15), monocytes (*CD14*^+^, cluster 4), so-called ‘non-classical monocytes’ (*CD14*^int^, *FCGR3A*^+^ encoding CD16, cluster 13)^40^ and MDSCs (*S100A8*^+^, *S100A9*^+^, *FCGR3*B^+^, cluster 10) (figure 3B, online supplemental figure 7A-7D and online supplemental table 4). Cluster 10 was also highly positive for the neutrophil signature^41^ and for a PMN-MDSC signature^29^ (online supplemental figure 7C-7F). These data therefore identified a complexity of macrophages and potential MDSCs in human tumors, as observed in the TH-MYCN model. Additional clusters of CAFs (*COL1A1*^+^, cluster 3), endothelial cells (*VWF*^+^, *ENG*^+^, cluster 6), cells expressing lymphatic endothelial markers (*LYVE1*^+^, *PROX*1^+^, *PDPN*^+^, cluster 17) and cycling cells (*MKI67*^+^, clusters 11 and 12) were also part of the TME (figure 3B and online supplemental table 4).

Regarding the macrophage populations, we observed that the different clusters expressed *CD68* and *APOE* (figure 3C, D) and were positive for a M2 signature but did not express the signature defining a M1 phenotype (online supplemental figure 7G-7H), which is in favor of a protumoral activity. Further analysis of signatures associated with an angiogenesis or phagocytosis phenotype highlighted cluster 9 and cluster 2, respectively (online supplemental figure 7I-7J). We noticed that *CSF1R* was expressed only in clusters 2 and 9 and *CD33* was not detected in clusters 8 and 9 (figure 3C, D). Cluster 2 specifically expressed *FOLR2* (figure 3D). Of note, a strong level of *FABP4,* known to be expressed in lung macrophages was observed in cluster 16 (online supplemental figure 7K). This is consistent with the observation that the TR5 sample contributing to this cluster corresponded to a lung biopsy (online supplemental table 1, online supplemental figure 6D). To further investigate the link between these different macrophage subsets and analyze lineage trajectories of the myeloid populations, we applied the scVelo tool to our RNA-seq data. As shown in figure 3E, the obtained trajectories suggested that macrophages of cluster 9 (APOE^+^CSF1R^med^) and cluster 8 (APOE^+^CSF1R^low^) are derived from APOE^+^CSF1R^+^ macrophages of cluster 2.

Since our cohort of 10 patient samples included tumors with and without *MYCN* amplification (5 of each) and tumors at diagnosis or at relapse (5 of each)(online supplemental table 1), we asked whether TME cell populations were different in these various categories. Interestingly, we observed that the proportions of MDSCs and non-classical monocytes were higher in the set of tumors with *MYCN* amplification compared with tumors without this abnormality (online supplemental figure 8A). Similarly, we noticed a higher proportion of MDSCs in tumors at relapse compared with tumors at diagnosis (online supplemental figure 8B). Of note, B cells and endothelial cells were also more abundant in terms of proportions in relapsed cases. These observations, although obtained on a small series of tumors suggest an accumulation of MDSCs in *MYCN*-amplified tumors, known to be aggressive tumors with a poor outcome and also in tumors studied at an advance stage of the disease.

### CAF heterogeneity in human neuroblastoma

Consistent with the CAF analyses done in murine neuroblastoma tumors we also addressed CAF heterogeneity in our cohort of human neuroblastomas. Analysis of known signatures^37^ identified one small subcluster expressing a CAF-S1 signature and a second subcluster was characterized by a strong CAF-S4 signature and was also highlighted by dVPL and imPVL signatures (online supplemental figure 9A). These observations are reminiscent of those obtained in the mouse TH-MYCN neuroblastoma model. As Seurat did not identify distinct CAF clusters, we further split the two subpopulations using specific signatures (online supplemental figure 9B). This enabled us to identify CAF-S1 and CAF-S4 clusters. A differential analysis of these two clusters identified upregulated genes in each cluster (online supplemental figure 9C-9D). Expression of CXCL12 and CCL2 cytokines was clearly detected in human CAF-S1 (online supplemental figure 9C).

### T cells in human neuroblastoma exhibit features of dysfunctional cells

**Figure 4:**
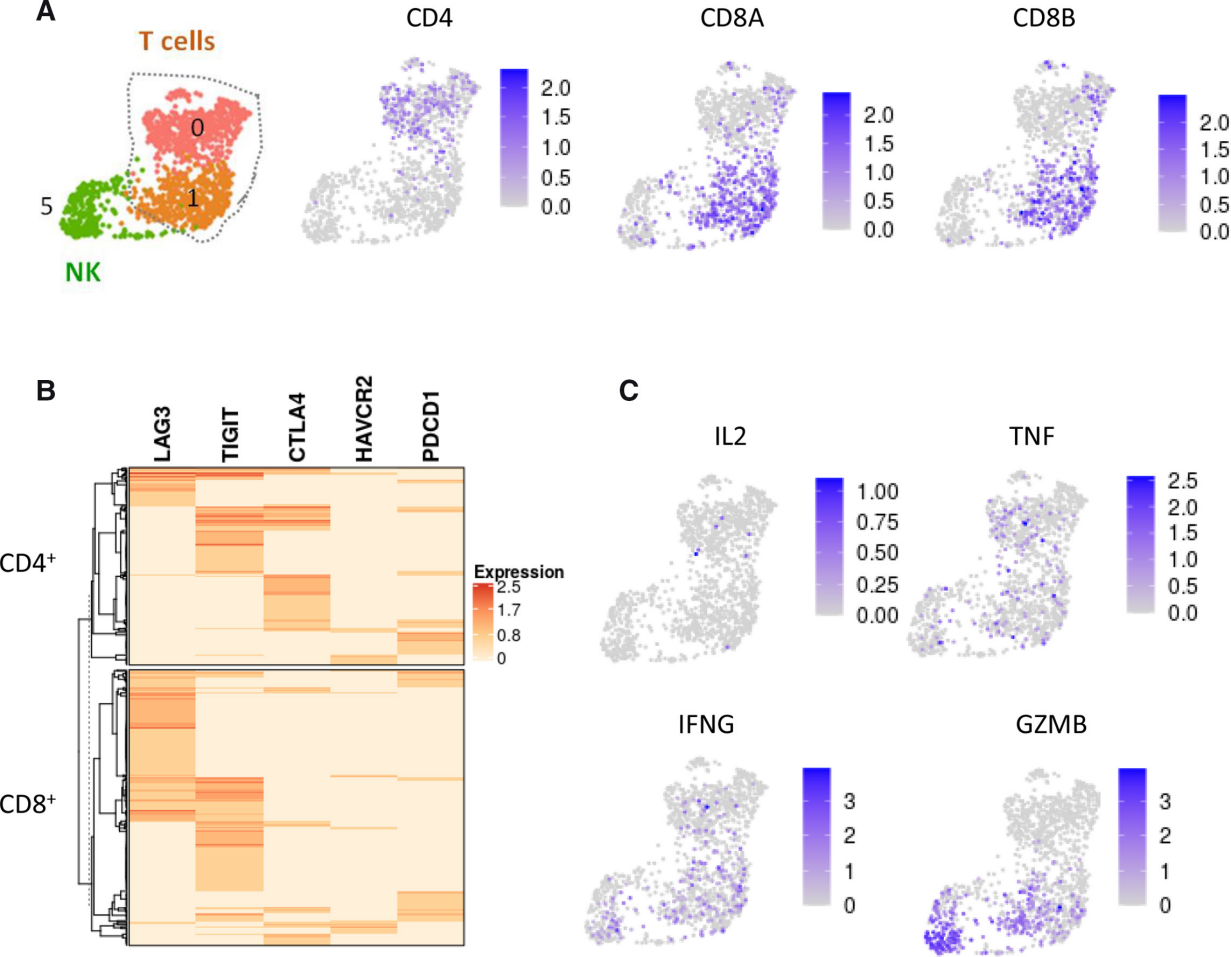
T cells in human neuroblastoma are dysfunctional. (A) Clusters 0 and 1 of T cells defined in figure 3A correspond to CD4^+^ and CD8^+^ cells, respectively. (B) Heatmap showing that all T cells express at least one inhibitory receptor. (C) Expression of the T cell effectors IL-2, TNF, IFNG and GZMB is absent or low in T cells.

Among immune cells, cytotoxic CD8^+^ T cells are one of the main effector cell types responsible for antitumor immunity. We noticed an important cluster of T cells in our cohort of 10 neuroblastoma patients, including CD4 and CD8 lymphocytes (figure 4A). It is now well described that immune suppressive agents in the TME lead to T-cell dysfunction, resulting in T-cell exhaustion.^42^ This state is characterized by increased expression of inhibitory receptors and decreased production of effector cytokines. Interestingly, our scRNA-seq data indicated that all T cells, being either CD4^+^ or CD8^+^, expressed at least one of the five well-described inhibitory receptors, being LAG3, TIGIT, CTLA4, HAVCR2/TIM3 and PDCD1/PD-L1 (figure 4B). A subset of CD8^+^ cells and few CD4^+^ cells co-expressed *LAG3* and *TIGIT*, whereas some CD4^+^ cells coexpressed *TIGIT* and *CTLA4*. Regarding effector cytokines, only sparse expression of *IL2*, *TNF*, *IFNG* and *GZMB* was observed in patient T cells (figure 4C). The expression profile of T cells infiltrating human neuroblastoma is therefore consistent with a phenotype of dysfunctional cells.

### Comparison of cell populations between MYCN-driven mouse neuroblastoma and human tumors reveals striking commonalities and a conserved population of MDSCs

**Figure 5:**
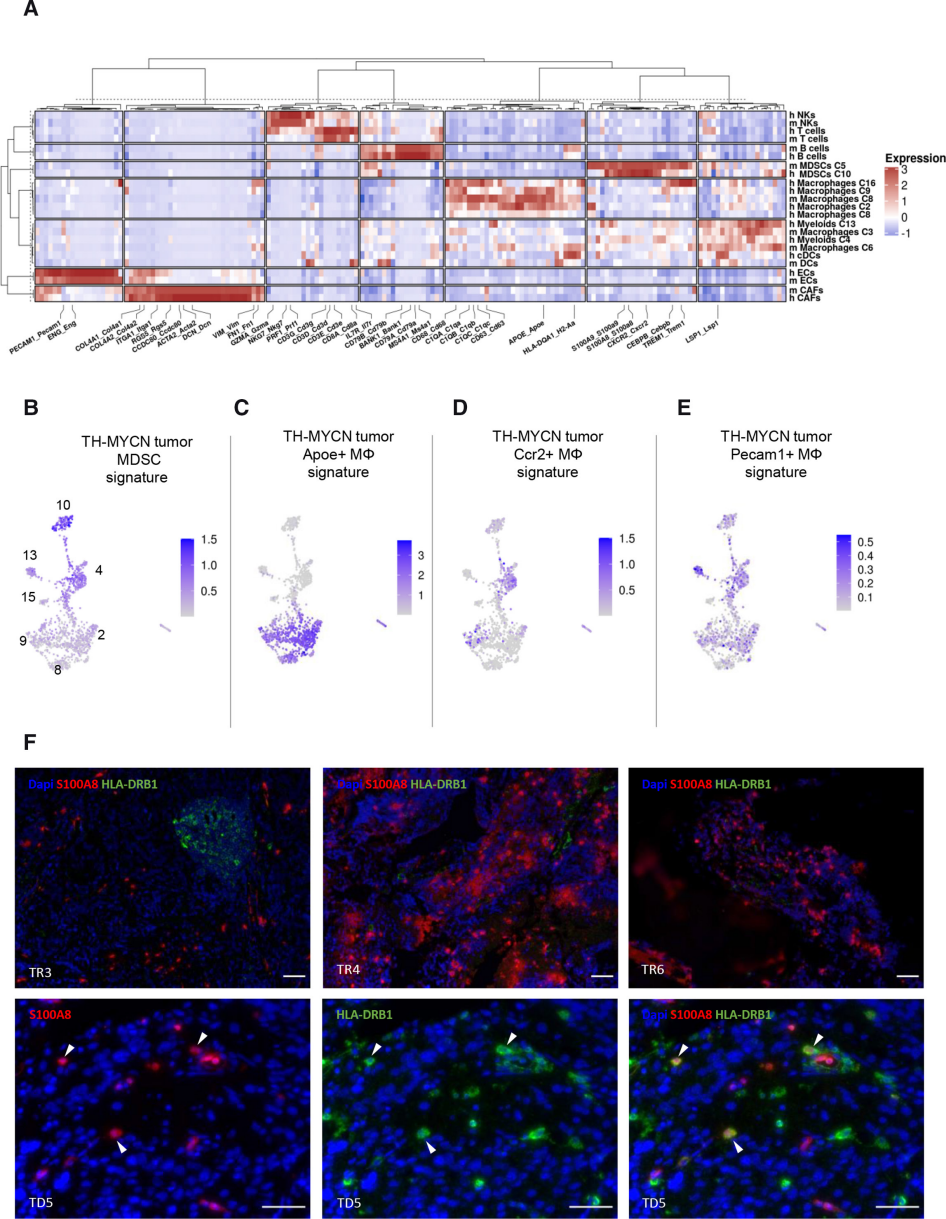
The myeloid-derived suppressor cell (MDSC) population identified in the mouse tumor microenvironment (TME) is conserved in human TME. (A) Hierarchical clustering using 169 genes selected as being upregulated in the main clusters of the mouse and human TME and presenting with a one-to-one orthologue. Each column represents a gene and each row corresponds to a cell population. (B) A signature including the top 20 genes upregulated in the mouse MDCS population (cluster 5) is strongly expressed in cluster 10 of the human TME. (C–E) Signatures of the three macrophage clusters defined in MYCN-driven mouse neuroblastoma are evaluated on the human myeloid cells of patient tumors. (F) Representative immunofluorescence images showing S100A8^+^ HLA-DRB1^-^ MDSCs in sections of microbiopsies from patients TR3, TR4 and TR6. S100A8^+^ HLA-DRB1^+^ corresponding to monocytes are shown in patient TD5 (arrows). Scale bar: 50 µm.

Having defined the TME heterogeneity both in mouse and human neuroblastoma, we further investigated similarities in population structure between both organisms. To do so, we first extracted the top 50 genes from each annotated clusters in both species, then got the average expression value of all genes in all clusters and finally performed an unsupervised hierarchical clustering of the genes and the different clusters. As shown in figure 5A, we observed that CAFs, endothelial cells, B cells, NKs and T cells of the two species clustered together indicating that their identity imposed the similarity between their gene expression profiles, rather than the analyzed organism. Among immune cells, the similarities in gene expression also reflected ontogeny with lymphoid cells separating from myeloid cells. Strikingly, a strong similarity between human cluster 10 and mouse cluster 5, previously identified as MDSCs, was observed. Common upregulated genes in both the murine and human populations included *S100A8*, *S100A9*, *CEBPB*, *CXCR2* and *TREM1* (figure 5A) as well as *HIF1A* and *PTGS2*/*COX2* (online supplemental tables 3 and 4) that have been recognized as features of MDSCs.^12^ These observations, therefore, highly suggested that human cells of cluster 10 are MDSCs with immunosuppressive activity. Our comparison highlighted complex relationships between the three populations of macrophages identified in the mouse model and the three identified in the patients. Indeed mouse cluster 8/Apoe^+^ macrophages clustered in a common branch with the three human macrophage clusters (figure 5A). Human clusters 4 and 13, annotated as non-classical monocytes and monocytes clustered together in a branch with murine Pecam1^+^ macrophages (cluster 3).

To confirm these findings, we next applied the mouse-derived gene signatures from myeloid cell populations in the human TME. Strikingly, a signature including the top 20 genes upregulated in the mouse MDSCs cluster defined in the TH-MYCN model was highly expressed in human cluster 10 and mildly expressed in human cluster 4 (figure 5B). The mouse Apoe^+^ macrophage (cluster 8) signature overlapped with all three macrophage clusters identified in patient tumors (figure 5C). In contrast, the murine Ccr2^+^ macrophage signature (cluster 6) rather highlighted human cluster 4 of monocytes (figure 5D). The murine Pecam1^+^ macrophage signature (cluster 3) mapped mostly with human cluster 13 of non-classical monocytes of the human samples (figure 5E). These observations are fully consistent with the aforementioned results of thehierarchical clustering.

Finally, to validate MDSCs in patient microbiopsies by an independent method, we performed immunofluorescence experiments using antibodies against S100A8 and HLA-DRB1 proteins. These two markers allow to distinguish MDSCs (cluster 10) that express S100A8 but not HLA-DRB1 from monocytes (cluster 4) that express both markers (figure 3B and online supplemental figure 7L).^35 43^ As shown in figure 5F, S100A8^+^ HLA-DRB1^-^ cells were observed in several cases, in agreement with our single-cell RNA-seq data.

Overall, our data identified a MDSCs population with high similarity between mouse and human neuroblastoma and revealed a high level of complexity in macrophage subsets from neuroblastoma consistently with recent data showing that macrophage subsets show species-specific patterns.^24 44^

### Immunosuppressive activity of MDSCs in the TH-MYCN model

**Figure 6:**
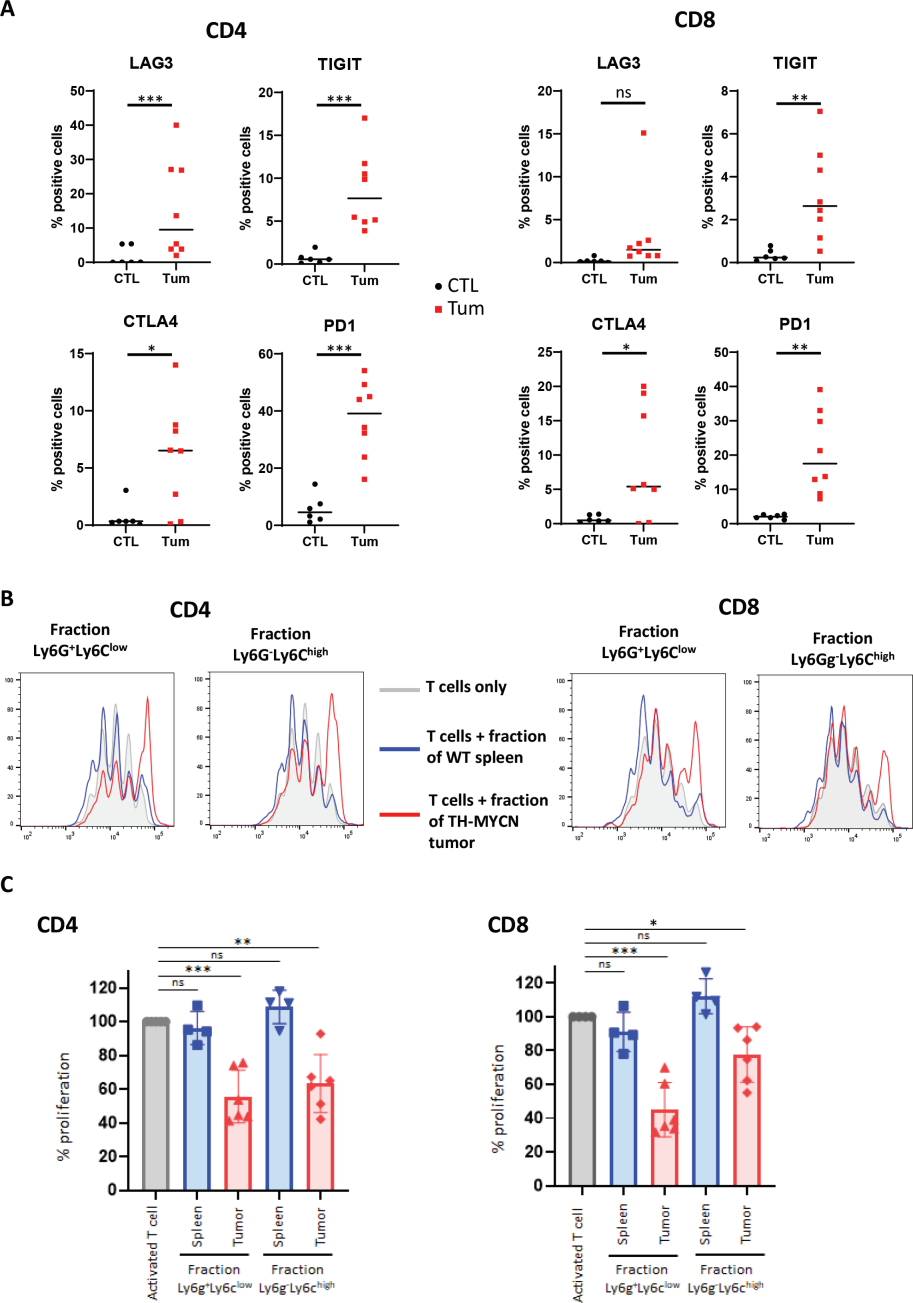
Exhausted phenotype of T cells and immunosuppressive activity of MDSCs from TH-MYCN mouse neuroblastoma. (A) FACS analysis showing that TH-MYCN neuroblastoma tumors exhibit more T cells expressing inhibitory receptors compared with spleen of wild-type mice. (B) CSFE proliferation profiles are shown for CD4^+^ and CD8^+^ cells after 3 days of coculture with CD45^+^CD11b^+^Ly6G^+^Ly6C^low^ cells or CD45^+^CD11b^+^Ly6G^-^Ly6C^high^ cells of a representative TH-MYCN tumor. The gray histogram corresponds to activated T cells only, the blue and red curves correspond to the histograms of T cells incubated with the two fractions purified from wild-type mouse spleen or TH-MYCN mouse tumor, respectively. (C) Functional assay demonstrating that CD45^+^CD11b^+^Ly6G^+^Ly6C^low^ cells and CD45^+^CD11b^+^Ly6G^-^Ly6C^high^ cells from TH-MYCN mouse tumors are able to inhibit T cell proliferation ex vivo. The same color code is used as in 6B. p-values: *:<0.05; **:<0.01; ***<0.001. CSFE, carboxyfluorescein succinimidyl ester; FACS, multicolor flow cytometry; MDSCs, myeloid-derived suppressor cells.

Since we observed that T cells of human tumors displayed increased expression of inhibitory receptors, we sought to analyze the phenotype of the rare T cells observed in the TME of TH-MYCN mouse tumors with respect to the expression of such receptors. FACS analysis using antibodies for LAG3, TIGIT, CTLA4 and PD1 indeed showed that more T cells significantly expressed these receptors compared with T cells obtained from spleens of wild-type mice (figure 6A), further demonstrating that anti-tumor response relying on T cells is impaired in the TH-MYCN mouse model.

MDSCs have been shown to exploit several mechanisms to modulate immune responses, since they can induce the proliferative arrest of antigen-activated T cells and restrain their function.^27 45^ To demonstrate the immunosuppressive property of MDSCs in neuroblastoma, we sought to perform functional assays. Such assays are extremely difficult to setup with human neuroblastoma cells. Indeed, only very limited number of tumor cells are available since patients are of very young age and tumor cells from high-risk patients are obtained in France only from small microbiopsies. Blood samples are also insufficient to obtain enough tumor-matched T cells from the same patient and using T cells from healthy donors would result in allogeneic immune responses. We therefore took advantage of the mouse model to perform immunosuppression assays and evaluate if mouse MDSCs present in TH-MYCN tumors are able to inhibit T cell functional activity. To do so, T cells purified from the spleen of wild-type syngenic mice and activated in vitro were incubated with PMN- or M-MDSCs isolated from mouse tumors by FACS sorting as CD45^+^CD11b^+^Ly6G^+^Ly6C^low^cells or CD45^+^CD11b^+^Ly6G^-^Ly6C^high^ cells. Similar cells purified from spleen of wild-type mice were used as a control. After 3 days of coculture, the proliferation of CFSE-labeled CD8^+^ and CD4^+^ T cells was assessed by FACS. This analysis showed that myeloid cells of the two fractions (CD45 +CD11b^+^Ly6G^+^Ly6G^low^ or CD45 +CD11b^+^Ly6G^-^Ly6C^high^) enriched from tumors significantly suppressed CD4^+^ and CD8^+^ T cell proliferation whereas the same fractions from wild-type spleens had no effect (figure 6B, C).

Altogether, our transcriptomic, IHC, FACS and functional data characterized a population of MDSCs in the TH-MYCN neuroblastoma model that exhibit immunosuppressive activity as demonstrated by the inhibition of T cell proliferation ex vivo and the exhausted state of T lymphocytes. Our data highly suggest that the corresponding population identified in human neuroblastoma exhibits immunosuppressive functions, contributing with macrophages to the malignant phenotype.

## Discussion

Our data, obtained by single-cell transcriptomics provide the first comprehensive analysis of mouse and human neuroblastoma microenvironment cells without any prior assumptions on surface markers. We could dissect the entire TME including endothelial cells, immune cells and CAFs, the identity of which could be unambiguously defined with canonical markers and signatures. Only one small cluster of the tumor mouse model (cluster 16) and one of the human TME (cluster 14) remained undefined. Importantly, our single-cell RNA-seq data of the mouse model, for which material can be easily obtained, were confirmed by FACS and IHC analyses for macrophages, T cells and MDSCs. Analysis of high-quality tumor material from young patients affected with cancer remains challenging. The parallel analysis of the TME of a mouse neuroblastoma model and a cohort of patients is one of the strength of our work and is particularly valuable to get insights into functional activity of some matched cell populations.

Both in the mouse neuroblastoma model and primary tumors, we document a high content of myeloid cells and further decipher the heterogeneity of these cells, with the description of several phenotypes of macrophages in both organisms and characterization of a population of MDSCs. We demonstrate that, in the TH-MYCN model, PMN-MDSCs are more abundant than M-MDSCs and that both types sorted from the murine model are able to inhibit T cell proliferation in an ex vivo functional assay. Of strong interest, is the similarity between the murine immunosuppressive MDSCs (cluster 5) and the cluster 10 of human cells in our hierarchical clustering exploring mouse and human cell clusters. Both populations express high levels of S100A8 and S100A9 that have been shown to greatly accumulate in MDSCs and are now recognized as one of the hallmarks of these cells.^12^ Consistently with RNA-seq data, S100A8^+^HLA-DRB1^-^ MDSCs could be detected in neuroblastoma patient microbiopsies. Our single-cell transcriptomic data clearly document that neuroblastoma MDSCs are molecularly distinct from the various subsets of macrophages described in both species.

Targeting MDSCs has been suggested as a therapeutic strategy to reverse immunosuppression and improve clinical outcome in cancer patients. Different approaches have been proposed to regulate MDSCs in tumors, such as reducing their recruitment, accumulation and suppressive functions.^12^ Our present results point out upregulated genes in such cells of the neuroblastoma TME that could orientate their targeting. Notably, our transcriptomic data reveal that *PTGS2*/*COX2* and *CXCR2* are among the most upregulated genes in both murine and human MDSCs. Cyclooxygenase 2 is one enzyme involved in the generation of prostaglandin E2, a product of lipid oxygenation that has been shown to accumulate in PMN-MDSCs and mediate the enhanced suppressive activity of such cells.^46^ The chemokine receptor CXCR2 has been shown to play a key role in the migration of MDSCs to tumors. Its inhibition by genetic ablation or using small-molecules inhibitors enhanced immunotherapy in several mouse models of cancer.^47 48^ MDSC depletion remains challenging since those cells have short-lifespan in tissues and are constantly replaced. Another approach may rely on MDSC reprogramming using inhibition of various pathways to enhance anti-tumor immunity, such as COX2 inhibition^49^ or treatment with all-trans retinoic acid (ATRA).^12^ Several studies pointed out that ATRA is able to promote the differentiation of M-MDSCs into macrophages and DCs and eradicated PMN-MDSCs in both tumor-bearing mice and patients.^12^ Retinoic acid-based therapeutics have been used in patients with neuroblastoma and some benefit has been reported through their ability to suppress tumor growth and promote cell differentiation.^50^ Yet, the potential effect of ATRA on tumor MDSCs has not been investigated. Finally, innovative therapies with genetically engineered myeloid cells delivering IL-12 have been recently used in a mouse model of rhabdomyosarcoma and demonstrated a proof of concept for reversing immunosuppression and activate anti-tumor immunity.^51^

Our data highlight the complex phenotypes of macrophages in the TME of both mouse and human samples. Whereas three subsets of macrophages could be defined in each organism, signatures of each cluster did not translate into one-to–one correspondence between the two species. These observations are fully consistent with results previously obtained on myeloid cells of adult non-small-cell lung cancer and on a mouse lung adenocarcinoma model documenting that macrophage subsets show species-specific patterns.^24 44^

We could document that the rare intratumor T cells of TH-MYCN neuroblastoma exhibited increased expression of inhibitory receptors. In our data of patient tumors, the correlations between the different populations defined by RNA-seq cannot be evaluated since only small microbiopsies have been analyzed and may not be representative of the full tumors when considered individually. Yet, our results show that despite their presence in human neuroblastoma, CD4^+^ and CD8^+^ T cells exhibit features of exhaustion with the expression of at least one inhibitory checkpoint. The link between exhaustion of tumor infiltrative T-cells and the presence or phenotype of MDSCs remains to be studied.

Our study also highlights different CAF populations both in mouse and human neuroblastoma TME. Although representing small populations in the tumor ecosystem, mouse CAF-S1 cells in the TH-MYCN model appear to be an important source of cytokines and may exert immunosuppressive functions as previously described.^36^ Patient tumors likely include also CAF-S1 and CAF-S4. Although the study of higher numbers of cells will be required to confirm our observations this first analysis allow us to identify CAF heterogeneity in neuroblastoma.

In conclusion, our present work has generated a new data resource through single-cell transcriptomics that provides a better understanding of the neuroblastoma ecosystem and the basis to develop both tumor-targeted and immune-targeted therapies. The degree of commonality between the TH-MYCN mouse model and our series of analyzed patient tumors indicates the relevance of using this animal model to evaluate the function of various populations in tumor progression and explore new immune therapeutic approaches.

## Data Availability

Single-cell RNA-seq data of the three TH-MYCN mouse tumors are available in Gene Expression Omnibus under the accession number GSE180101. Single-cell RNA-seq data from patients are available in European Genome-Phenome Archive (EGA) under the accession number EGAS00001004781 (ongoing submission).
