# Supplementary material for: Single-cell transcriptomics reveals shared immunosuppressive landscapes of mouse and human neuroblastoma
Source: J Immunother Cancer. 2022 Aug 5;10(8):e004807. doi: 10.1136/jitc-2022-004807 (PMC9362821; doi:10.1136/jitc-2022-004807)
Supplement: Supplementary data [file jitc-2022-004807supp002.pdf]

SUPPLEMENTARY METHODS

TH-MYCN mouse model

Mice on a 129×1/SvJ background were obtained from the NCI mouse repository (http://mouse.ncifcrf.gov/) and further backcrossed on 129S2/SvPasCrl background (abbreviated 129S2, Charles River). Genotyping was performed as previously described [1]. All analyzed mice were heterozygous for the transgene. Bearing tumor mice for the single-cell RNA-seq analysis (42369, 41884 and 42007) were studied at 62, 79 and 63 days, respectively. The care and use of animals used in this study were strictly applying European and National Regulation in force for the Protection of Vertebrate Animals used for Experimental and other Scientific Purposes (Directive 86/609). Approval for this study was received from Ministère de l'Education Nationale, de l'Enseignement Supérieur et de la Recherche (authorization number 5524-20 160531 1607151 v5).

Tumor dissociation into single-cell suspension

Mouse tumors and patient samples (biopsies and surgical resections) were cut with scalpels in small fragments. Enzymatic dissociation was performed in GIBCO medium containing 150 µg/mL Liberase™ TL Research Grade (5401020001, Merk) and 150 µg/mL DNase (DN25, Sigma Aldrich), for 30 minutes at 37°C with 400 rpm agitation. Cell suspension was then filtered using a 70 µm cell strainer (130-098-462, Miltenyi Biotec). The cell suspension was washed twice with PBS. Viability was measured using Vi-cell XR Viability Analyzer (Beckman Coulter).

Single-cell RNA-seq experiment and preprocessing of data

Single-cell RNA-seq was performed with the 10x Genomics Chromium Single Cell 3' Kit (v3.1) according to the standard protocol. Libraries were sequenced on an Illumina HiSeq2500 or NovaSeq 6000 sequencing platform. CellRanger version 3.1.0 (10x Genomics) was used to demultiplex, align and generate UMI count tables from sequencing reads. Two reference genomes were used to align reads:

the mouse reference genome (mm10) for the 3 MYCN-driven mouse tumors; the human reference genome (hg38/GRCh38) for the 10 patient samples.

Ambient mRNA correction

SoupX R package v1.4.5 (https://github.com/constantAmateur/SoupX) was used to estimate and correct for ambient mRNA contaminations in both mouse and patient tumors. First, depending on the expression profile of empty droplets (defined as droplets with UMI count <100), we selected, as recommended, either *HBB/HBA* or immunoglobulin genes (*IGHA1, IGHA2, IGHG1, IGHG2, IGHG3, IGHG4, IGHD, IGHE, IGHM, IGLC1, IGLC2, GLC3, IGLC4, IGLC5, IGLC6, IGLC7, IGKC*) as marker genes to estimate contamination fractions. When these genes were absent from the ambient profile (the top 100 covered genes), we used the automatic mode provided by SoupX to estimate contamination fractions and generate corrected expression matrices. Summary of analyses is shown in Supplementary Tables 5 and 6.

Doublet detection

Scrublet v0.2.1 [2] was used to detect potential doublets using default parameters (expected_doublet_rate=0.06). Cells marked as doublets were removed from subsequent analysis (results in Supplementary Tables 5 and 6).

Quality control of single-cell data

First, all ribosomal genes (defined as *RLP/RPS* genes) were removed from the raw expression matrices. Then, coverage thresholds were set for each sample individually; an upper threshold was set to remove outlier cells with coverage greater than the 99th percentile, and a lower threshold was set to remove low quality cells with coverage inferior to the 1st percentile. To avoid cells with low number of genes, the same lower threshold was applied on the number of genes thus defining a minimum

number of genes required. Finally, cells with more than 20% of reads mapping mitochondrial genes were removed.

Normalization of single-cell data

Raw UMI counts were normalized using the “SCTransform” function of Seurat v3.1.5 [3]. Regressed variables included cell coverage, number of features, and the percentage of UMI from mitochondrial genes.

Dimensionality reduction and cluster identification

Normalized count data was subjected to dimensionality reduction keeping the first 30 principal components. Uniform Manifold Approximation and Projections (UMAP) embeddings were calculated using these Principal Components (PCs) as input and cells were clustered using the “FindClusters” function of Seurat. We also generated an umap using the graph structure produced by “FindNeighbors” on which “FindClusters” function detects cell clusters. This umap representation is referred to as “umap.graph” in Supplementary Figure 6.

Cell type annotation

Marker genes that define cell clusters were identified after differential expression analysis using Seurat “FindAllMarkers” function. Clusters were annotated by comparing their top marker genes to canonical cell type markers from the literature. Additional cell type annotation was performed using singleR v1.0.6 [4] which annotates cells against built-in references datasets. We used the Human Primary Cell Atlas [5] and data from the Immunological Genome Project [6] to annotate cells in the human and mouse micro-environment, respectively.

Generation of single-cell signature scores

To plot the expression of gene signatures in single cells, we used the “AddModuleScore” function from Seurat R package with 100 genes in the control gene set.

scRNA-seq data integrations

Seurat v3.1.5 was used to integrate the 3 TH-MYCN mouse tumors and the 10 neuroblastoma patient samples using 3,000 anchor features. The integrated objects were subjected to dimension reduction and clustering as described above. Tumor cells and microenvironment were identified based on the expression of specific marker genes. To further study human TME, 3,785 non-tumor cells were extracted from the integration. Raw counts were first normalized using SCTransform function (Seurat) then Harmony v1.0 [7] was used for data integration.

Copy number analysis

Copy number variations at the single cell level were called with R package InferCNV v1.2.1 [8] (https://github.com/broadinstitute/inferCNV) using default parameters. Cells with fewer than 1000 UMI were excluded and normal cells from the microenvironment of one patient were used as reference cells (n=733).

RNA velocity

To perform RNA velocity analysis [9], we first used velocyto CLI to annotate spliced /unspliced reads from cell-barcodes sorted bam files. We then used the scVelo package [10] with default parameters and default data preparation procedures to compute steady-state velocities and visualize velocity streamplots on myeloid cell populations.

Immunohistochemistry (IHC)

Formalin-fixed paraffin embedded (FFPE) tumors from TH-MYCN mice were cut in sections (4 µm) and prepared for staining using standard protocols for xylene and alcohol gradient for deparafination (Sakura, Tissue-Tek DRS). All stainings were performed in the Lab Vision IHC stainer Autostainer 480 device (Thermo Scientific). The epitope retrieval was performed in EnVision FLEX Target Retrieval Solution low-pH (Dako, #K800521, for CD3 and S100A8) or high-pH (Dako, #K800421, for F4/80) followed by 5 minutes blockade of endogenous peroxidase activity with Dako REAL peroxidase-blocking solution (Dako, #S202386). A blocking step was made using Protein Block (Dako, #X-0909) for 10 min. The tissue sections were then incubated with the F4/80 (Abcam, #Ab6640; 1:2000), CD3 (Dako, #A0452; 1:200) or S100A8 primary antibody (ThermoFisher, #PA5-79948; 1:100) or Rabbit IgG isotype control (Abcam, #Ab172730) for 1 hour at RT, followed by wash with 1X PBST (Dako, #K8000). Next, a goat anti-rabbit antibody (VECTOR laboratories, #PK-6101 kit) was incubated for 25 minutes at RT for CD3 and S100A8 whereas a rabbit anti-rat antibody was used for F4/80 (Cliniscience, #BA-4001). The tissue sections were washed using PBST and then signal detection was performed by incubation with avidin-horseradish peroxidase (Vector Laboratories) for 25 minutes and detected with 3,3'-diaminobenzidine for 5 min (DAB, Dako, #K3468). Counterstaining was performed with Mayer hematoxylin freshly prepared (Dako, #S3309). Tissue sections were then submitted to serial gradients of xylen and mounted with coverslip in an automatic device (Sakura, Tissue-Tek DRS). Staining overview of the sections was done in the Zeiss Axioplan microscope and slides were then scanned using the Philips Ultra Fast Scanner and visualized at high resolution in the Philips IMS 2.2 software for further analyses and photo acquisition.

FACS analysis

Following tumor digestion described in "*Tumor dissociation into single-cell suspension section*", 2x10^6 cells per sample were stained with Aqua Live/Dead viability dye (Life Technologies) according to the manufacturer's instructions. Cells were stained with an antibody panel for

immunophenotyping (see Supplementary Table 7 for a list of antibodies, clones, fluorochromes, manufacturers, and concentrations) for 30 minutes at RT in dark. After staining, cells were washed in PBS supplemented with 2 mM EDTA and 0.5% FBS. Data was acquired with a BD™ LSRII flow cytometer and further analyzed using Flowjo v10. For the lymphoid panel, $CD19^+$ B cells were gated as $CD45^+/CD19^+$, $CD3^+$ T cells as $CD45^+/CD3^+$ and $CD19^-CD3^-$ cells correspond to myeloid and NK cells (Supplementary Figure 10).

For the analysis of T cell inhibitory receptors, cell suspensions obtained from TH-MYCN mouse tumors as described above were pre-enriched in $CD45^+$ cells by magnetic beads (130-110-618, Miltenyi Biotec) according the manufacturer's instructions. CD45+ cells were suspended in buffer (PBS, 1%SVF, 5mM EDTA) and incubated with CD4-eF450, CD8-APC, CD279 (PD1)-PC7, CD152 (CTLA4)-AF700, TIGIT-PE and CD223 (LAG-3)-PerCP-eF710 antibodies for 30 min at 4°C in dark. After staining, cells were washed in PBS. Data were acquired with a BD LSRII flow cytometer and analyzed using Flowjo v10 for the expression of the inhibitory receptors in CD4+ and CD8+ cells.

Mouse-human comparison heatmap

For an unsupervised comparison between mouse and human microenvironment cell populations, only genes with one-to-one translation between human and mouse were considered, as previously defined [11]. Starting from the two integrations (human and mouse TME), we extracted the top 50 marker genes of each cell population. These markers were filtered for mitochondrial and cell cycle genes. We then retained the intersection between human and mouse marker genes to define the final set of genes used for the inter-species comparison (n=169). The average expression value of these genes within all cell populations was computed and scaled independently within each specie. Finally, we combined the scaled average expression values for human and mouse into a single expression table and used ComplexHeatmap R package [12] for visualization. To better highlight expression patterns, we partitioned the heatmap into 7 sub-groups using k-means clustering on rows

(selected marker genes) and columns (cell populations). Additional complete hierarchical clustering with Pearson correlation distance was applied to further cluster rows and columns within each sub-group.

Co-immunofluorescence on paraffin-embedded tissues

Paraffin-embedded tissue blocks were cut into 5 μm sections. Immunostaining was processed in a Bond RX automated (Leica) with Opal™ 7-Color IHC Kits (Akoya Biosciences, NEL821001KT) according to the manufacturer's instructions using antibodies anti-HLA Class II DRB1 (Abcam mouse monoclonal, ab212448, 1/4000[e], 30min - Opal520) and anti-MRP8/S100A8 (Abcam rabbit monoclonal, ab92331, 1/2000[e], 60min - Opal690) Tissue sections were coverslipped with Prolong™ Diamond Antifade Mountant (Thermo Fisher). Slides were scanned using the Vectra® 3 automated quantitative pathology imaging system (Vectra 3.0.7; Akoya Biosciences). Multispectral images were unmixed using the inForm Advanced Image Analysis Software (inForm 2.6.0; Akoya Biosciences).

MDSCs isolation from TH-MYCN mouse tumors and mouse WT spleens

Tumors were harvested from TH-MYCN mice and mechanically dissociated with a scalpel. Enzymatic dissociation was performed as described above. To enrich for viable cells, we performed a cell debris removal kit (130-109-398, Miltenyi Biotec) according the manufacturer's instructions. Spleens of wild-type mice were crushed on a 70 µm cell strainer (130-098-462, Miltenyi Biotec). Cell suspensions were washed twice with 1X PBS. Viability was measured using Vi-cell XR Viability Analyzer (Beckman Coulter). Both for tumors and spleens, cell suspensions were pre-enriched in $Cd45^+$ cells using magnetic beads (130-110-618, Miltenyi Biotec) according the manufacturer's instructions and were resuspended in buffer (PBS supplemented with 1% SVF and 5 mM EDTA). Cell suspensions were incubated for 30 minutes at 4°C in dark with pre-conjugated fluorescent labeled with the following combination: CD45-APC-Cy7, CD11b-FITC, Ly6G-APC (127614, Biolegend), and Ly6C-Alexa700

antibodies. Flow cytometry sorting was performed with the SH800S cell sorter (Sony). The first gating was based on FSC/SSC. Doublet cells were eliminated by gating on SSC-W/ SSC-H followed by FSC-W/FSC- H. The second gating was based on DAPI negative staining to eliminate dead cells. $CD45^+/CD11b^+/Ly6G^+/Ly6C^{low}$ (PMN-MDSC) cells and $CD45^+/CD11b^+/Ly6G^-/Ly6C^{high}$ (M-MDSCs) were sorted.

T cell suppression assay

Cell suspensions from spleens were obtained as described above. T cells were isolated using the mouse Pan T Cell Isolation Kit II (130-095-130, Miltenyi Biotec) according to the manufacturer's instructions. T cells (up to 10^7) were stained using the CellTrace™ CSFE Yellow Cell Proliferation Kit (C34573, Thermofisher Scientific) at 5 µM for 8 minutes at 37°C. The staining was stopped by incubation with 5 volumes of PBS supplemented with 5% heat-inactivated FBS for 10 minutes at 4°C. Finally, T cells were washed twice with complete medium (RPMI Glutamax) containing 10% heat-inactivated FBS, 1% Penicillin-Streptomycin, 0.1% β-mercaptoethanol, 1% Non-Essential Amino Acid supplement, 1% Hepes, 1% Sodium Pyruvate) at 4°C. Labeled T cells (10^5/well) were plated and activated in complete medium with anti-CD28 (553294, BDBiosciences, 1 µg/mL) in a flat bottom 96-well plate previously coated with anti-CD3 (Clone 145-2C11, BDBioscience CD3e, 10 µg/mL, diluted in 1X PBS). Sorted $CD45^+/CD11b^+/Ly6G^+/Ly6C^{low}$ cells and $CD45^+/CD11b^+/Ly6G^-/Ly6C^{high}$ cells from TH-MYCN tumors or WT spleens were added to the T cells using a 10:1 T cells:MDSC sorted cells ratio. After 3 days of culture, cells were collected and stained with fluorescent-conjugated antibodies CD4-PE-Cy7 (BLE100528, Biolegend, 1/100) and CD8-APC (553035, BDBioscience, 1/100) for 25 minutes at 4°C, protected from the light. Cells were washed and incubated with DAPI followed by flow cytometry analysis performed with the BD™ LSRII cytometer. The percentage of proliferation was calculated with the following formula [Y (T cells alone] / Y (T cells + MDSC sorted cells)] X 100. The Y value corresponds to the mean fluorescent intensity of CFSE of the whole T cell population divided by the mean fluorescent intensity of CFSE of undivided T cells.

References

1. Haraguchi S, Nakagawara A. A simple PCR method for rapid genotype analysis of the TH-MYCN transgenic mouse. PLoS One. 2009;4:e6902.

2. Wolock SL, Lopez R, Klein AM. Scrublet: Computational Identification of Cell Doublets in Single-Cell Transcriptomic Data. Cell Syst. 2019;8:281-291.e9.

3. Stuart T, Butler A, Hoffman P, Hafemeister C, Papalexi E, Mauck WM, et al. Comprehensive Integration of Single-Cell Data. Cell. 2019;177:1888-1902.e21.

4. Aran D, Looney AP, Liu L, Wu E, Fong V, Hsu A, et al. Reference-based analysis of lung single-cell sequencing reveals a transitional profibrotic macrophage. Nat Immunol. 2019;20:163–72.

5. Mabbott NA, Baillie JK, Brown H, Freeman TC, Hume DA. An expression atlas of human primary cells: inference of gene function from coexpression networks. BMC Genomics. 2013;14:632.

6. Heng TSP, Painter MW, Immunological Genome Project Consortium. The Immunological Genome Project: networks of gene expression in immune cells. Nat Immunol. 2008;9:1091–4.

7. Korsunsky I, Millard N, Fan J, Slowikowski K, Zhang F, Wei K, et al. Fast, sensitive and accurate integration of single-cell data with Harmony. Nat Methods. 2019;16:1289–96.

8. Tickle T, Tirosh I, Georgescu C, Brown M, & Haas B. inferCNV of the Trinity CTAT Project. 2019;

9. La Manno G, Soldatov R, Zeisel A, Braun E, Hochgerner H, Petukhov V, et al. RNA velocity of single cells. Nature. 2018;560:494–8.

10. Bergen V, Lange M, Peidli S, Wolf FA, Theis FJ. Generalizing RNA velocity to transient cell states through dynamical modeling. Nat Biotechnol. 2020;38:1408–14.

11. Zilionis R, Engblom C, Pfirschke C, Savova V, Zemmour D, Saatcioglu HD, et al. Single-Cell Transcriptomics of Human and Mouse Lung Cancers Reveals Conserved Myeloid Populations across Individuals and Species. Immunity. 2019;50:1317-1334.e10.

12. Gu Z, Eils R, Schlesner M. Complex heatmaps reveal patterns and correlations in multidimensional genomic data. Bioinformatics. 2016;32:2847–9.
