# Supplementary material for: Single-cell transcriptomics reveals shared immunosuppressive landscapes of mouse and human neuroblastoma
Source: J Immunother Cancer. 2022 Aug 5;10(8):e004807. doi: 10.1136/jitc-2022-004807 (PMC9362821; doi:10.1136/jitc-2022-004807)

Supplementary Figure 1

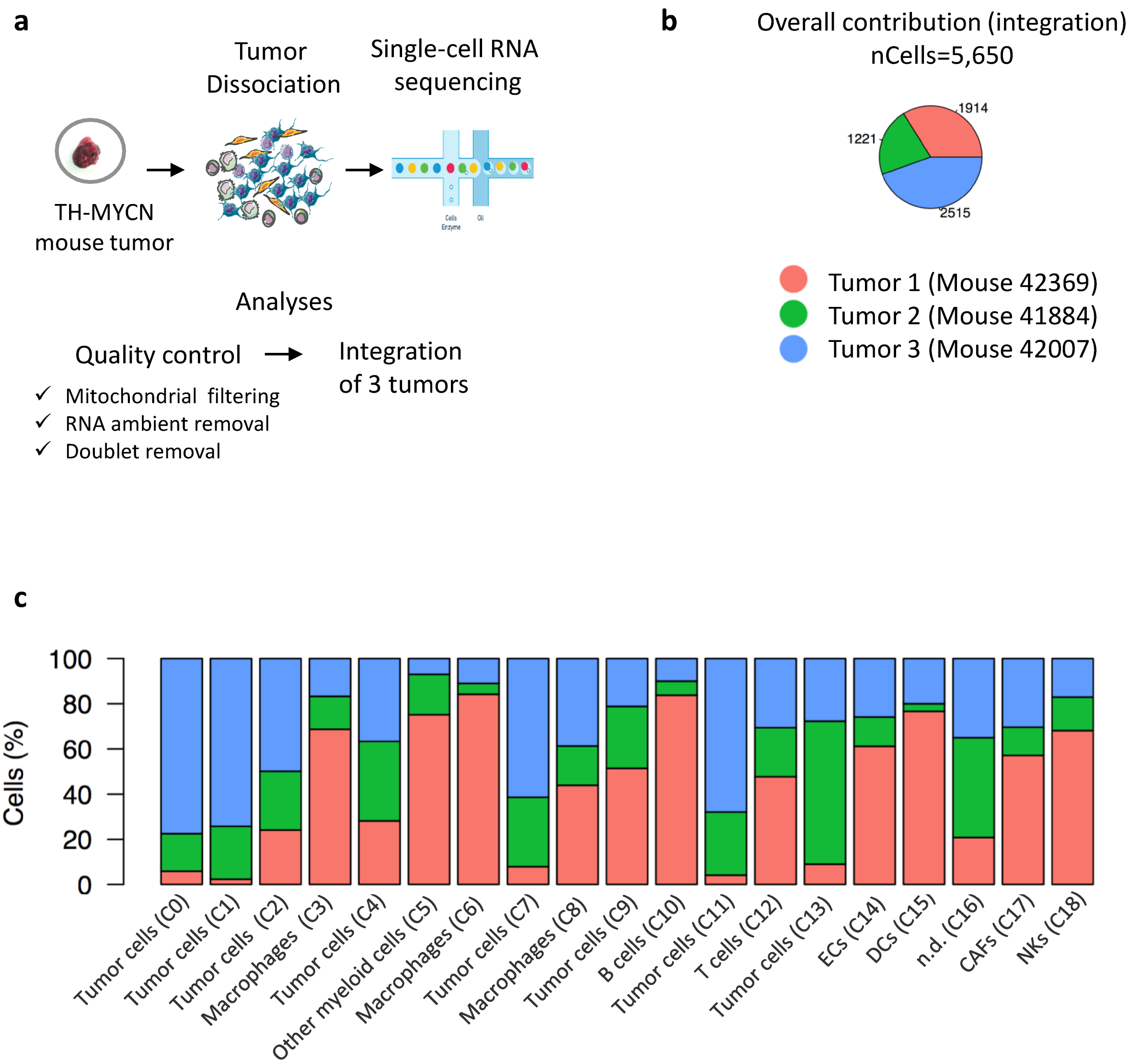

Supplementary Figure 2

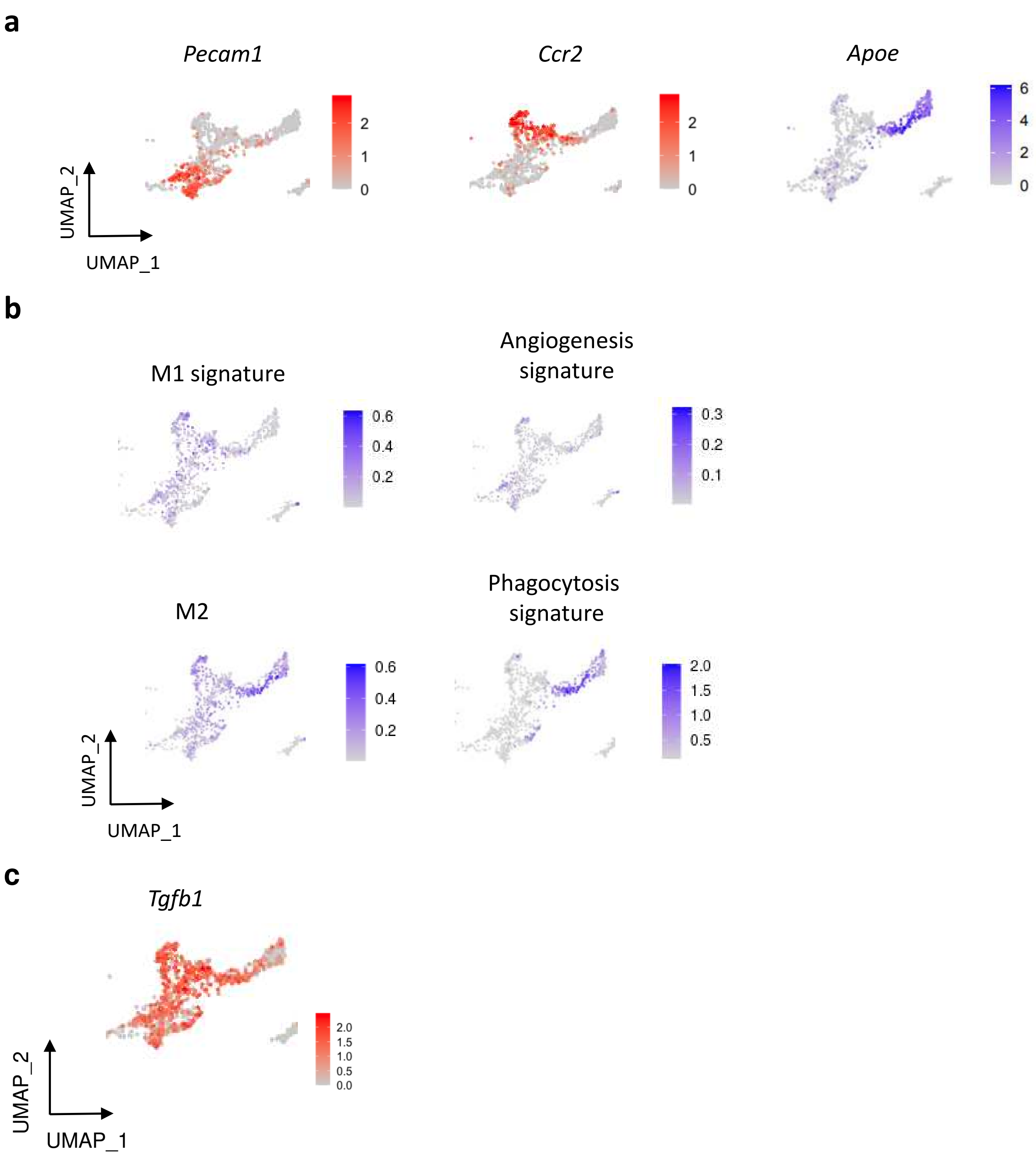

Supplementary Figure 3

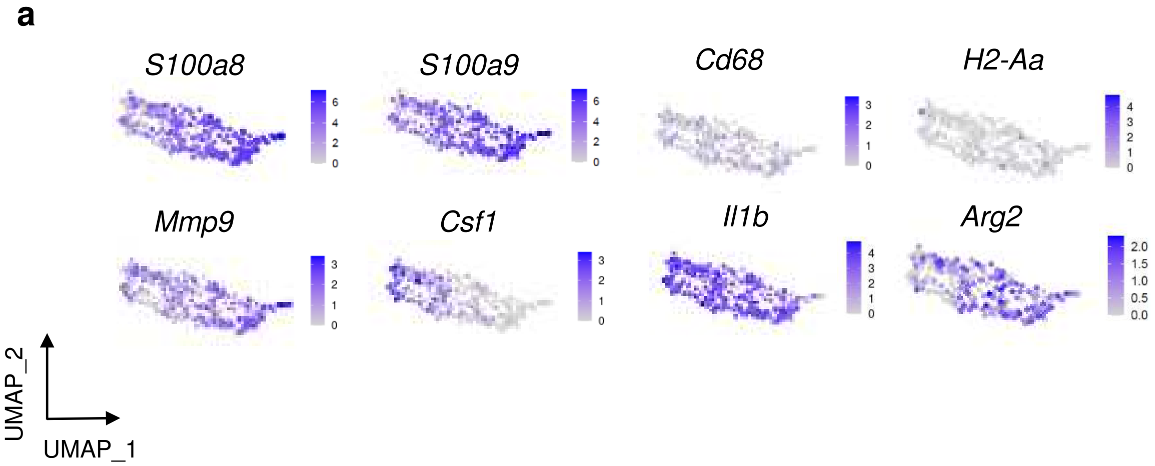

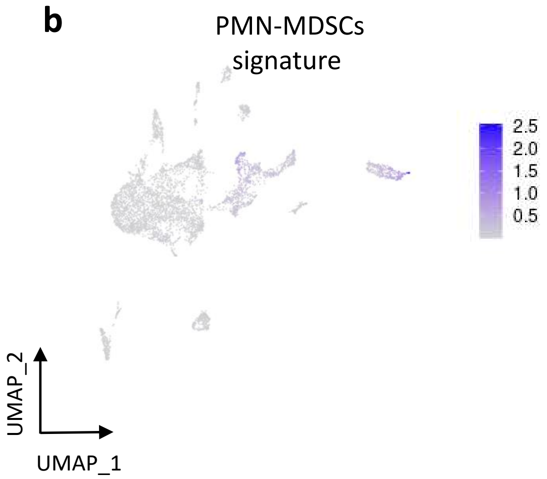

Supplementary Figure 4

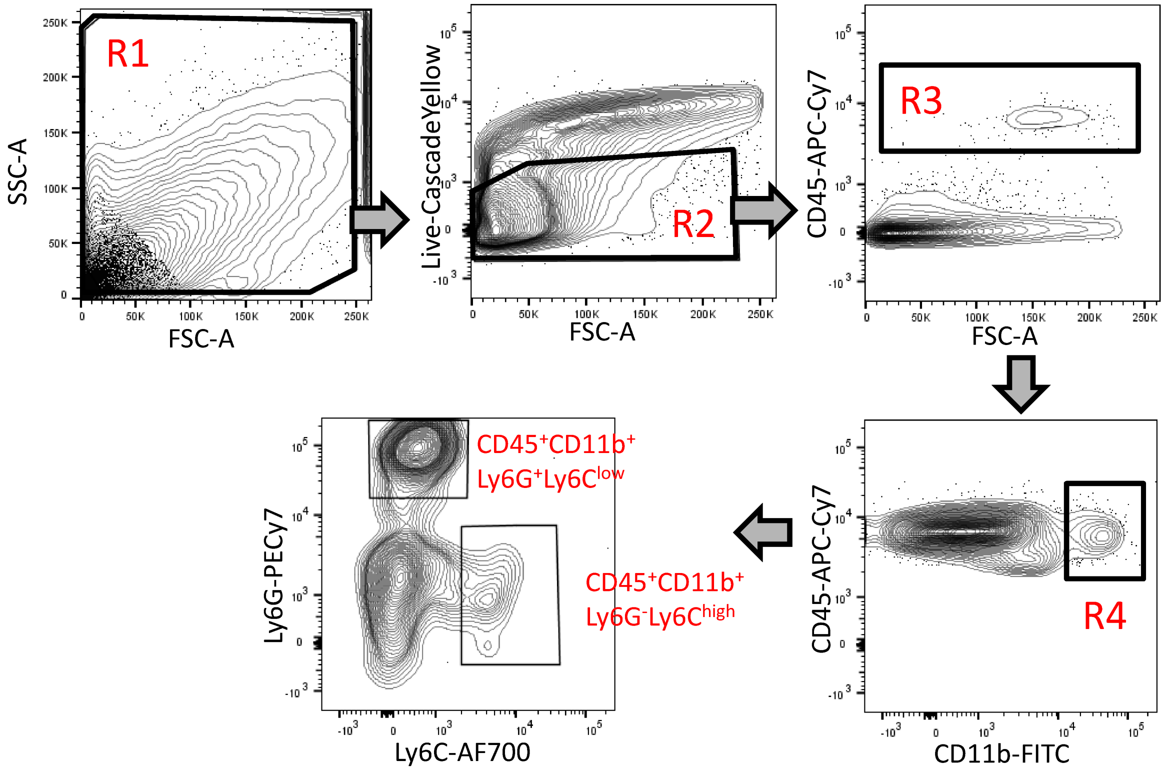

Supplementary Figure 5

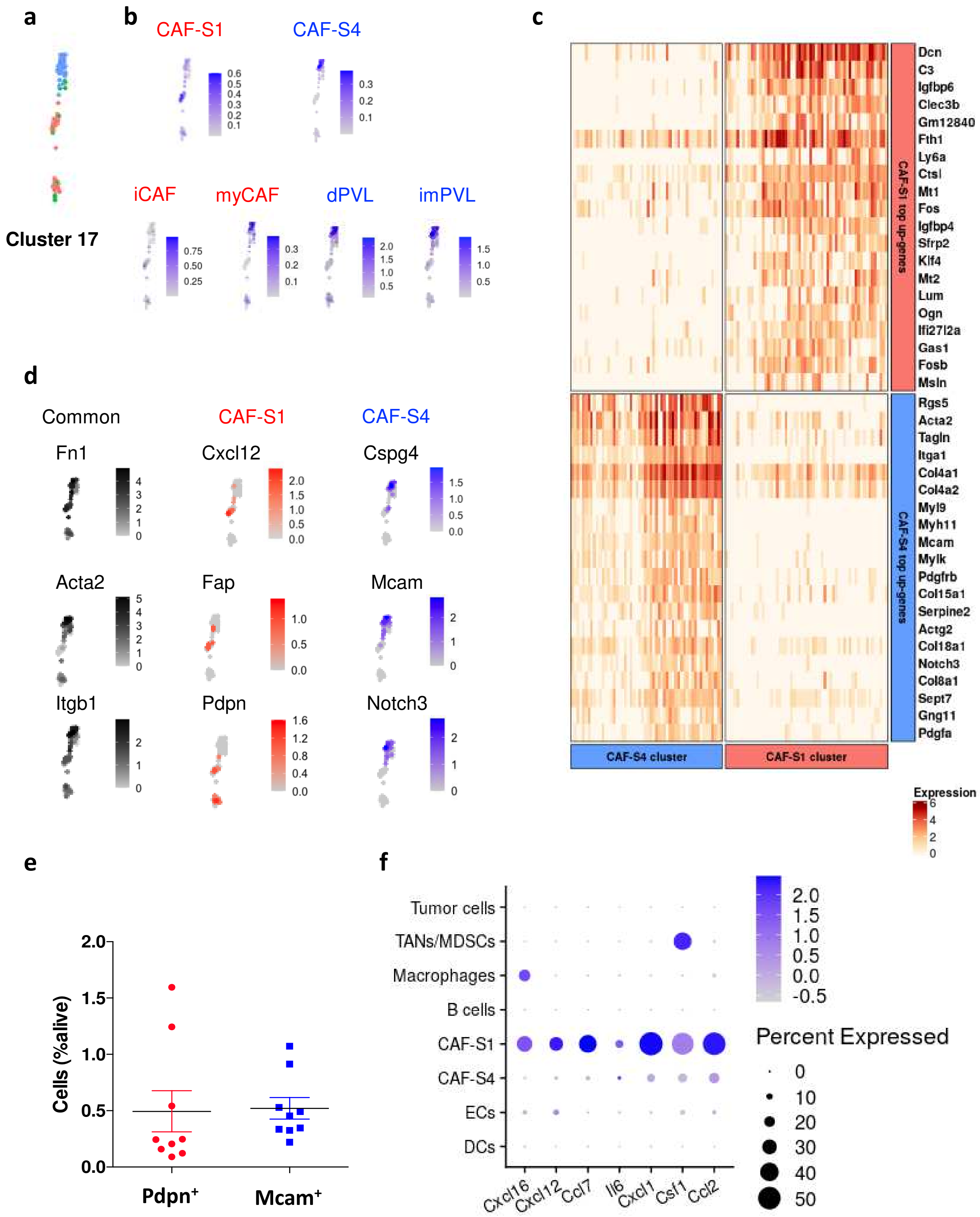

Supplementary Figure 6

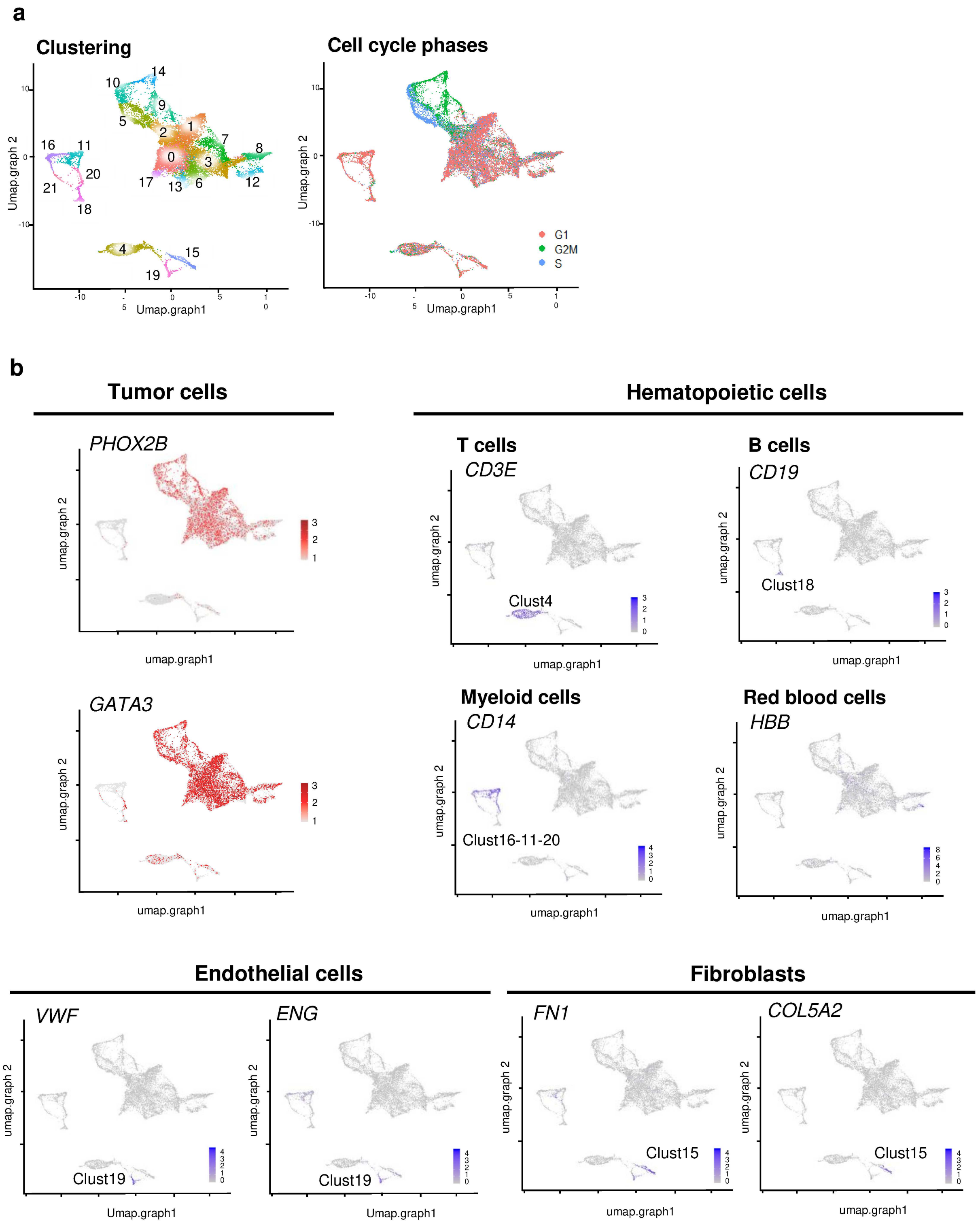

Supplementary Figure 6

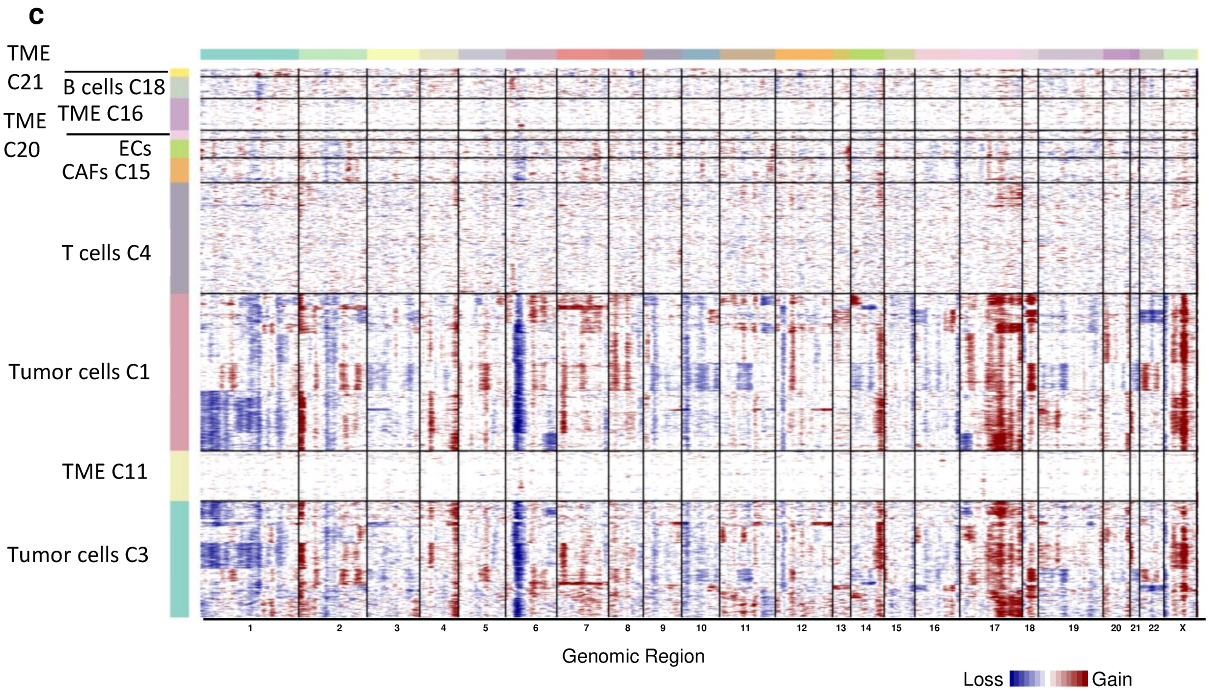

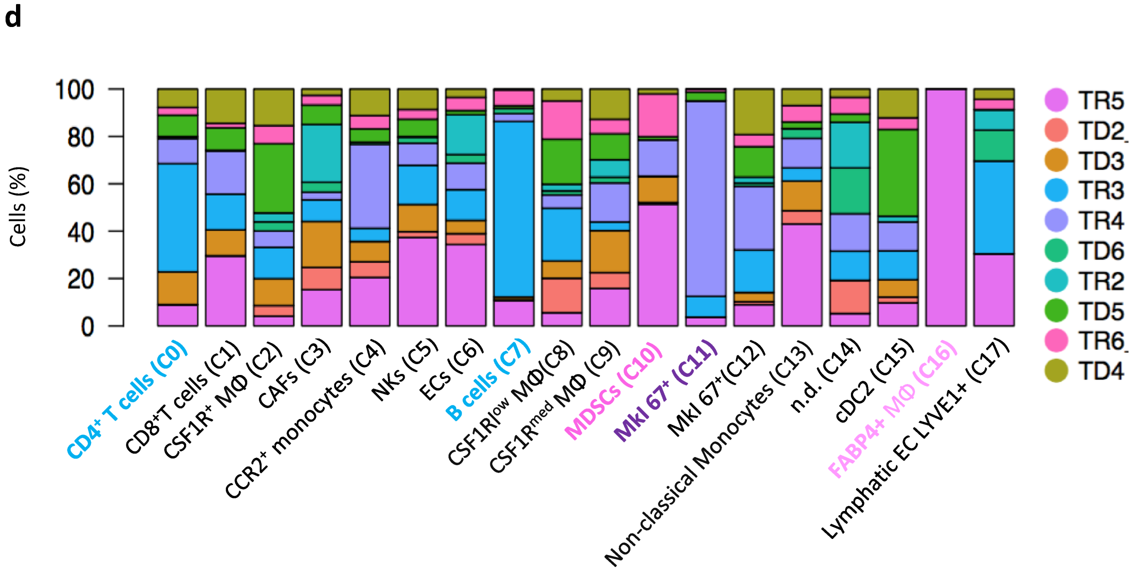

Supplementary Figure 7

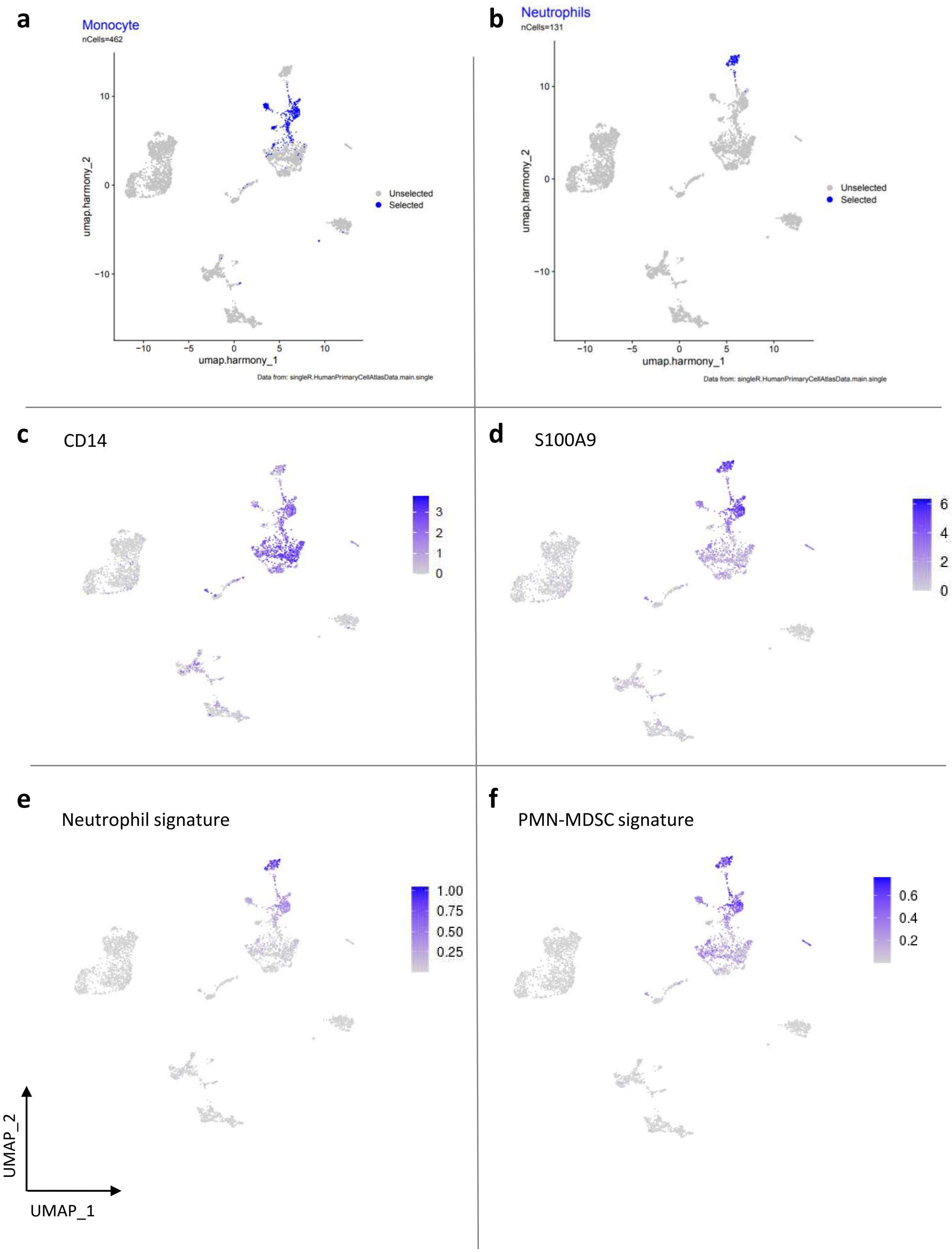

Supplementary Figure 7

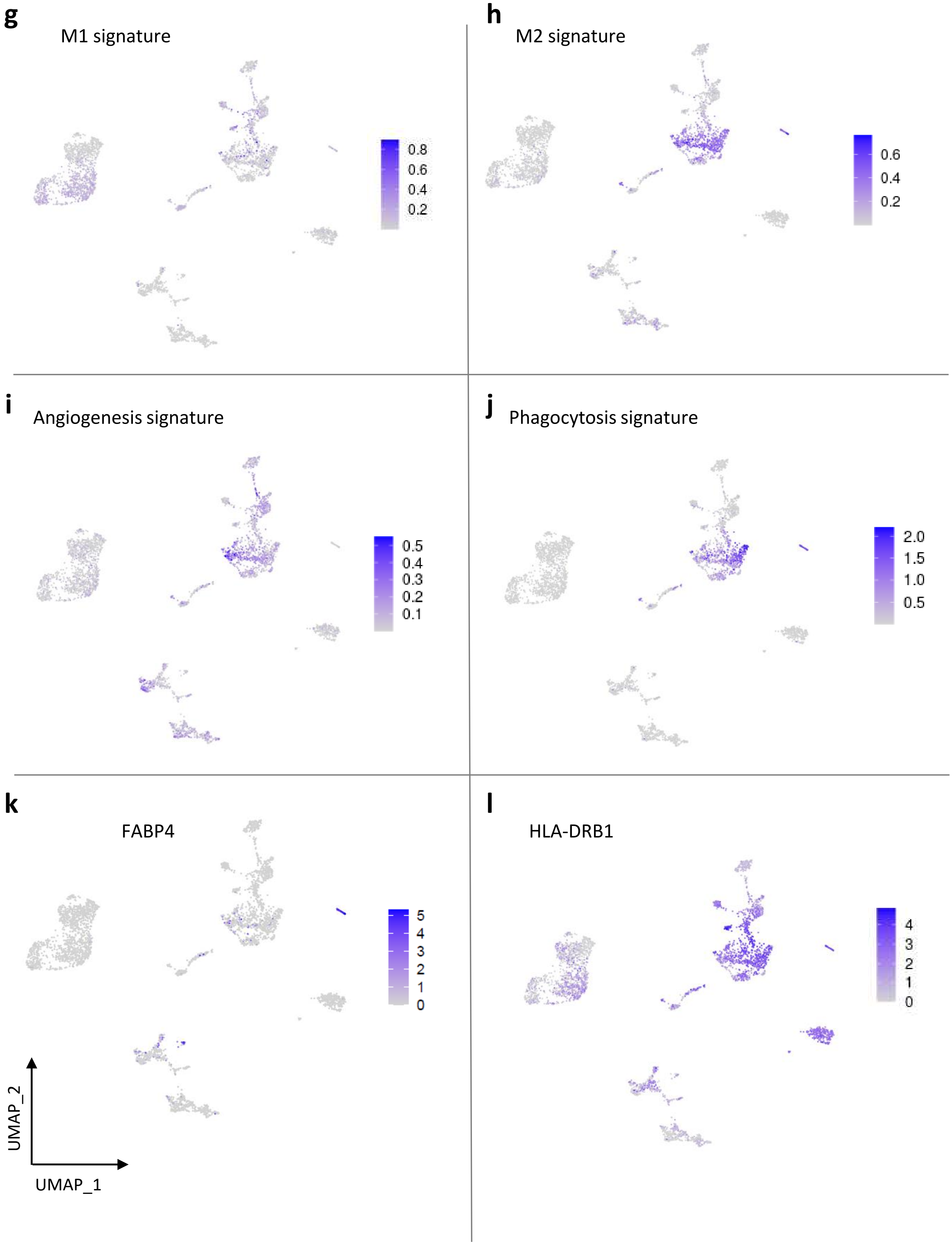

Supplementary Figure 8

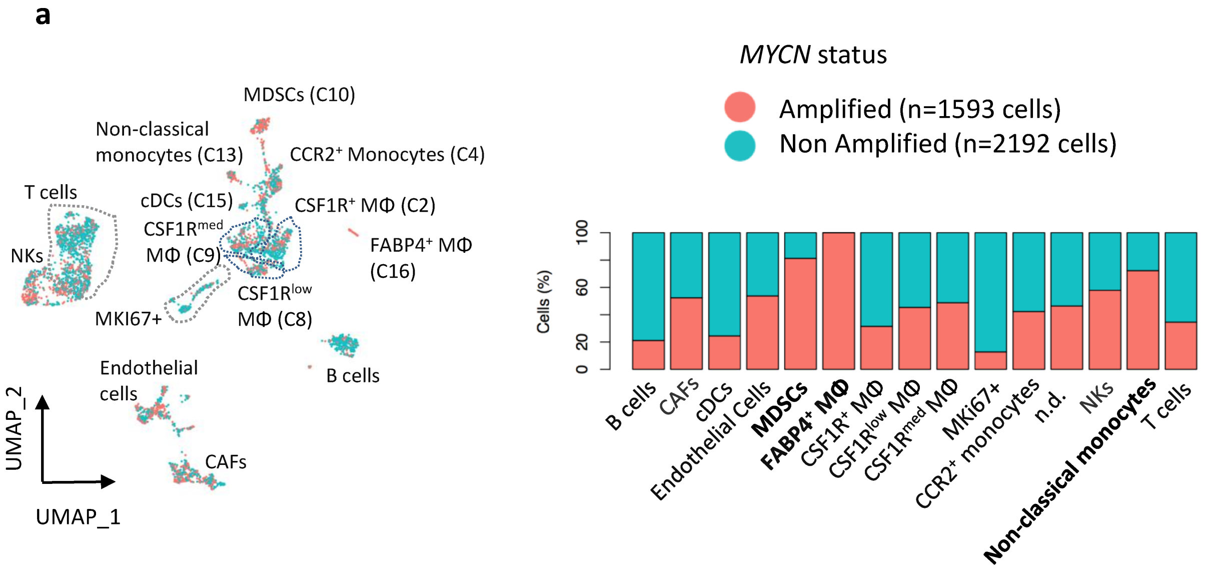

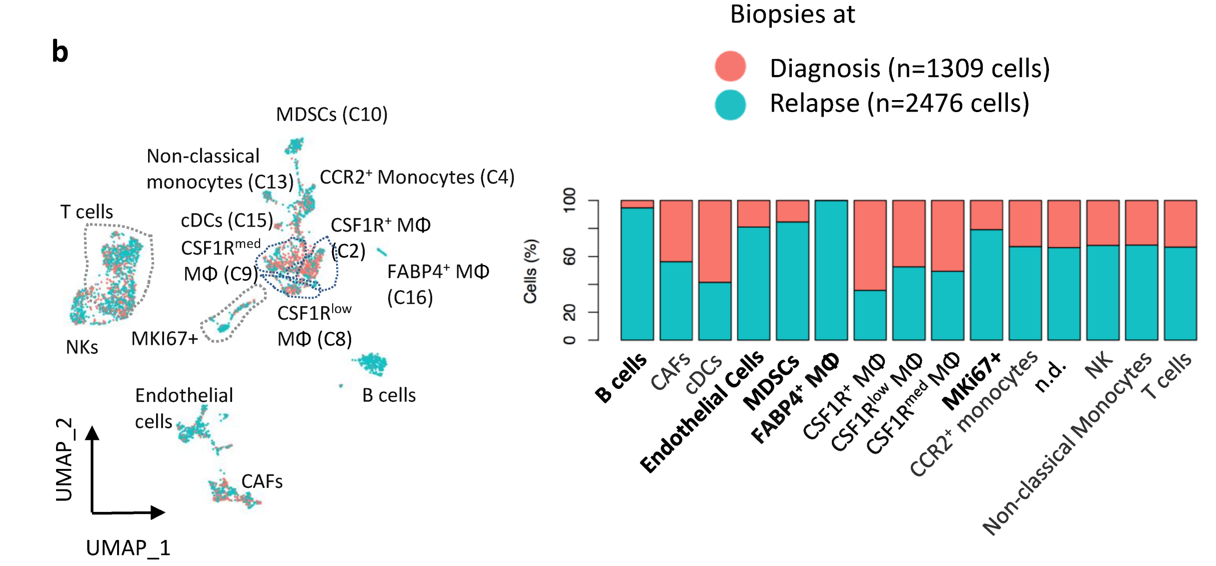

Supplementary Figure 9

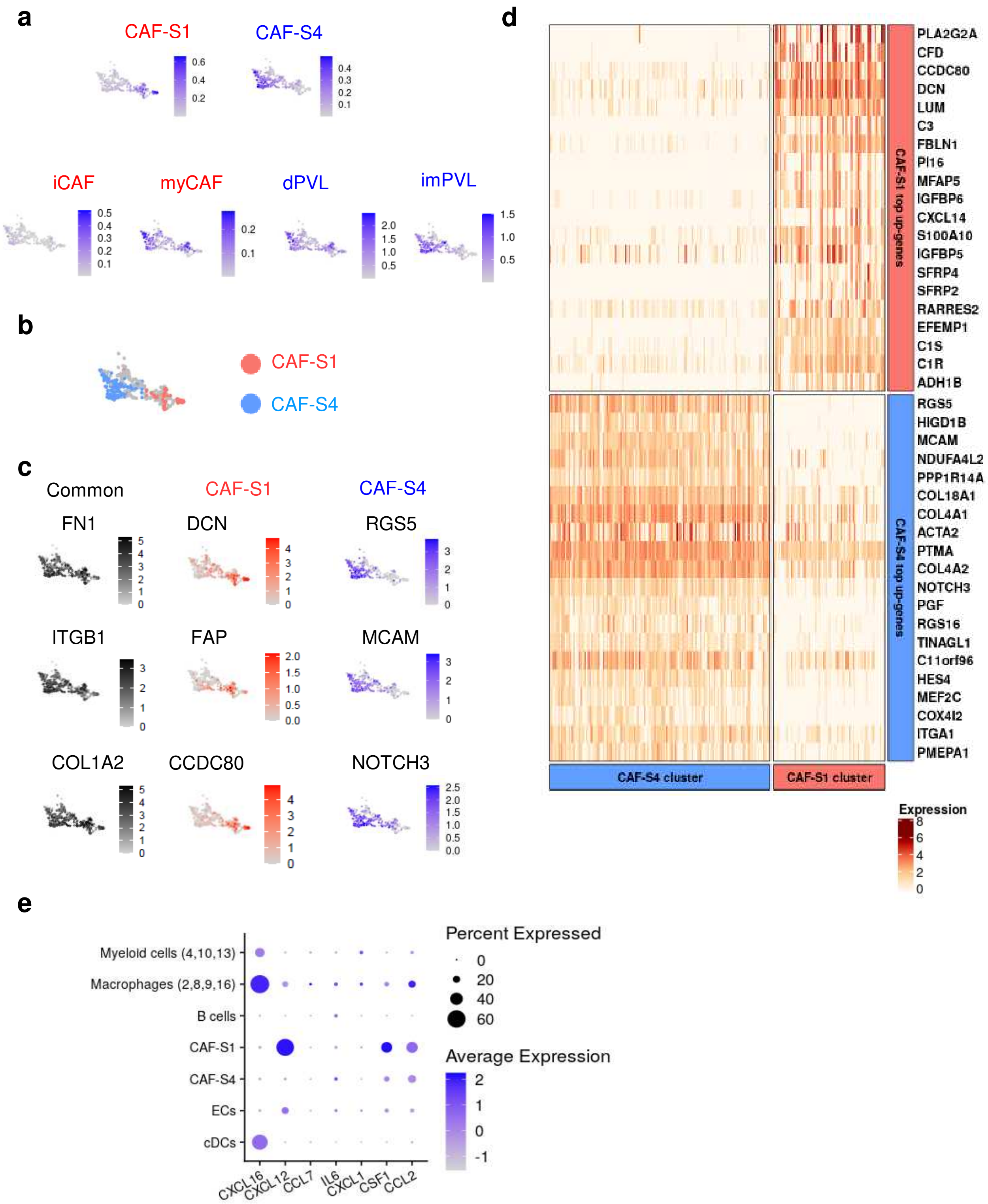

Supplementary Figure 10

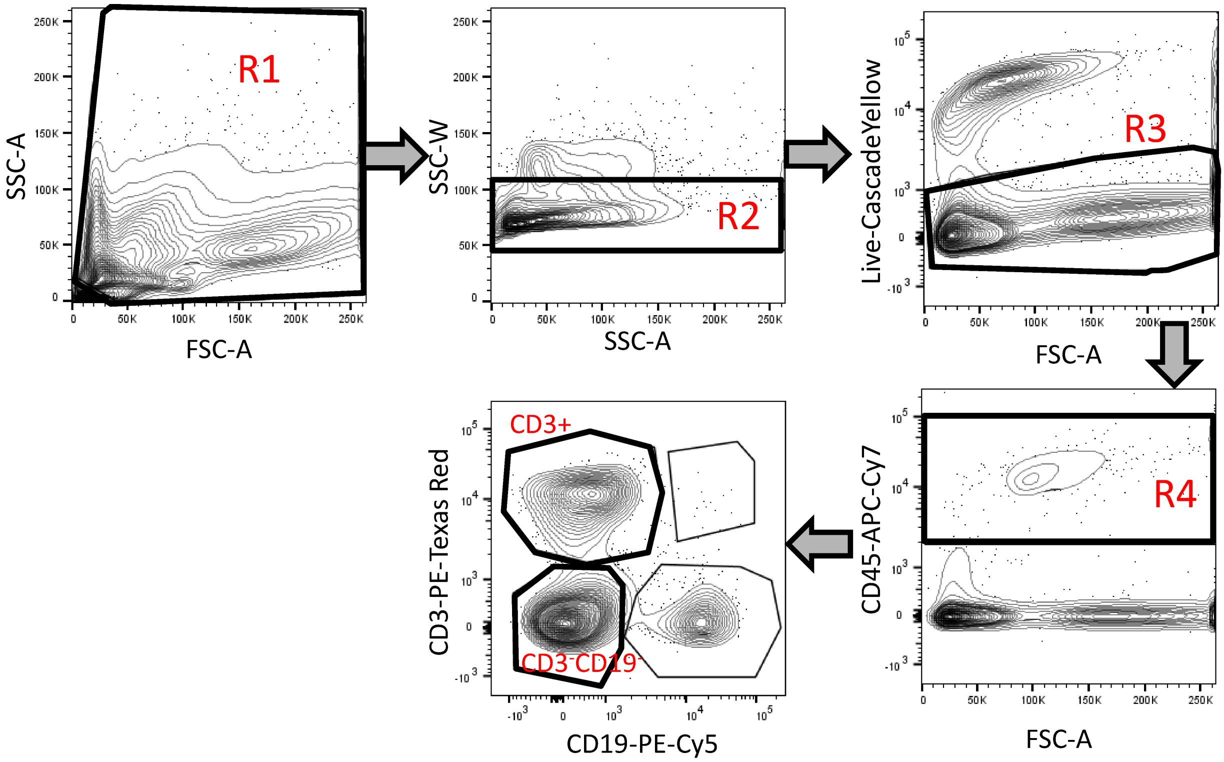

Supplement: Supplementary data [file jitc-2022-004807supp001.pdf]
